# Longitudinal assessment of the association between pesticide exposure and lifestyle with Parkinson’s disease motor severity

**DOI:** 10.1038/s41531-025-01010-2

**Published:** 2025-06-12

**Authors:** Theresa Lüth, Amke Caliebe, Carolin Gabbert, Sebastian Sendel, Björn-Hergen Laabs, Inke R. König, Christine Klein, Joanne Trinh

**Affiliations:** 1 Institute of Neurogenetics, University of Lübeck, Lübeck, Germany; 2 Institute of Medical Informatics and Statistics, Kiel University and University Hospital Schleswig-Holstein, Kiel, Germany; 3 Institute of Medical Biometry and Statistics, University of Lübeck, Lübeck, Germany

**Keywords:** Risk factors, Signs and symptoms, Parkinson's disease

## Abstract

Longitudinal investigations on the relationship between lifestyle exposures and motor severity are lacking. In this longitudinal study, we included patients with idiopathic Parkinson’s disease (iPD) (*N* = 5139) and *LRRK2*-related PD (*N* = 81) from PPMI-Online and Fox Insight. Motor aspects were followed for up to five years. We investigated the association between environmental exposure, lifestyle factors and motor aspect severity over time by applying linear mixed effects models. In *LRRK2*-PD, black tea consumption was associated with less severe motor aspects (β = −0.51, *p* = 0.028). In patients with iPD, pesticide exposure was associated with more severe motor aspects over time in PPMI-Online (β = 0.23, *p* = 3.56 × 10^−^^9^). Lastly, caffeinated soda was associated with more severe motor aspects in patients with iPD from PPMI-Online (β = 0.15, *p* = 3.84 × 10^−8^) and Fox Insight (β = 0.09, *p* = 0.031). We suggest that pesticide exposure and lifestyle factors may affect motor severity in patients with *LRRK2*-PD and iPD, demonstrating the impact on patients even after disease onset.

## Introduction

PD is a progressive neurodegenerative disorder that currently affects close to 12 million patients worldwide^1,2^. From 1990 to 2021, the age-standardized number of individuals with PD increased by 60.7%; at the same time, the number of Alzheimer’s disease patients increased only by 3.2%^2^. As the number of cases has risen constantly over the last decade, PD will be a large burden to society, especially since there is no cure available and only medication to manage the signs and symptoms of the disease. Among the ~15% of PD patients where the disorder is explained by a single pathogenic variant (i.e., monogenic PD) or strong risk factor^3,4^, the LRRK2 G2019S variant is the most frequent cause of autosomal-dominant PD (*LRRK2*-PD). Recently, a 3.5-year longitudinal study assessed disease severity in patients with *LRRK2*-PD in the 23andMe dataset, and it has been suggested that patients present predominantly with PD motor feature impairment and have less severe non-motor features compared to patients with idiopathic PD (iPD)^5^.

Besides genetics, the environment and lifestyle are of great importance in PD. Pesticide exposure is one of the most compelling environmental risk factors for PD^6,7^ and is associated with an earlier age at onset (AAO)^8,9^. Additionally, there has been recent evidence that pesticides impact PD patients after disease onset, as a longitudinal study showed that exposure is associated with faster disease progression^10,11^. The associations between PD risk and other environmental exposures, such as air pollution^12–14^, solvents^15–17^, and heavy metals^15,18–21^ have also been explored, but partially with variable outcomes. Smoking and coffee are the most crucial lifestyle factors associated with reduced PD risk^6^ and are analogously associated with later AAO, as demonstrated by many studies^6,22–26^. With regard to monogenic *LRRK2*-PD, smoking, coffee and black tea consumption are associated with a later AAO as well^26,27^. Interestingly, caffeinated soda, on the other hand, was found to be associated with earlier AAO in *LRRK2*-PD^26^.

Longitudinal studies examining the relationship between smoking, caffeine, and motor aspect severity are underexplored. In contrast to the proposed protective effect of smoking on PD susceptibility and onset, a cross-sectional assessment suggested an association between smoking and more severe motor and non-motor features in PD^28^. This observation highlights that different mechanisms might mediate the impact of environment and lifestyle before and after disease onset. Thus, a more thorough and longitudinal analysis is required to elucidate environmental and lifestyle factors’ association with PD severity over time. Results from longitudinal cohort studies provide a higher evidence level when compared to case-control studies, which suffer from recall bias, retrospective study design, enhanced confounding and lack of differentiation between pre- or post-disease exposures. Since genetic and environmental modifiers of onset have been identified for *LRRK2*-PD, such as black tea drinking, smoking and gene-environment interactions^26,29,30^, we focused our study on *LRRK2*-PD. In addition, distinguishing between *LRRK2*-PD and iPD will allude to a better understanding of the natural history of the different PD subtypes and possibly also the specific treatment responses.

Large available PD cohorts, including multiple patient assessments over the years, are an essential resource for investigating motor severity over time. Fox Insight is one of the largest cohorts of such kind, and participants are followed up for five years^31^, as well as Parkinson’s Progression Markers Initiative Online Study (PPMI-Online), in which patients are followed up for over three years^32,33^.

**Fig. 1:**
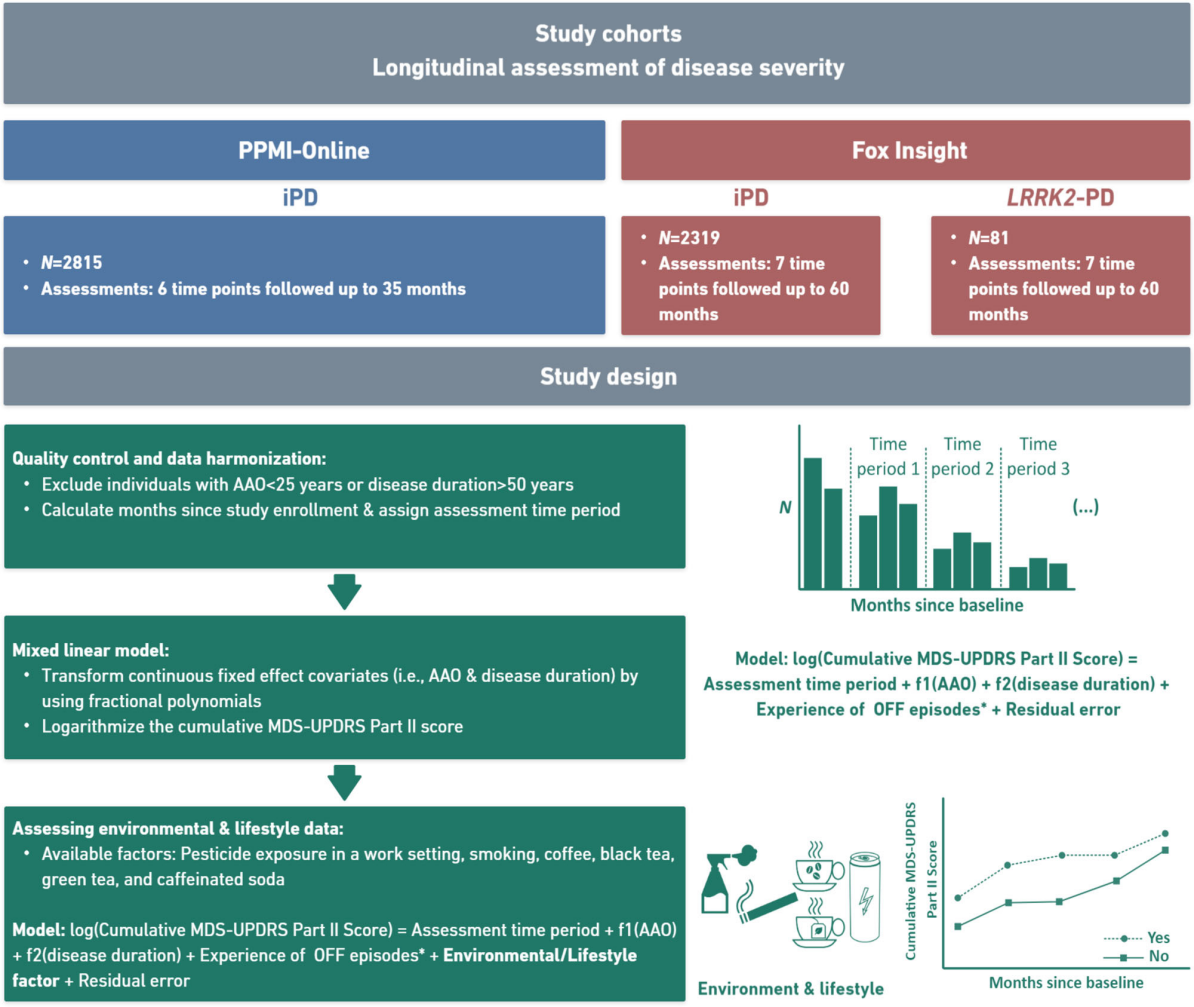
Study overview. The diagram illustrates the included longitudinal cohorts and the study design (data quality control, construction of the statistical model and overlaying of environmental/lifestyle data). *N* number of individuals, iPD Patients with idiopathic Parkinson’s disease, and *LRRK2*-PD Patients with PD who carry the LRRK2 p.Gly2019Ser variant, f1/f2 polynomial transformation age at onset or disease duration. *If applicable.

Therefore, we investigated motor aspects longitudinally in patients with *LRRK2*-PD from Fox Insight and patients with iPD from PPMI-Online and Fox Insight. Herein, we focused on the association of environmental and lifestyle factors (i.e., pesticide exposure, smoking and caffeine consumption) and motor aspect severity over time (Fig. 1).

## Results

### Motor aspect severity over time in iPD and LRRK2-PD

**Fig. 2:**
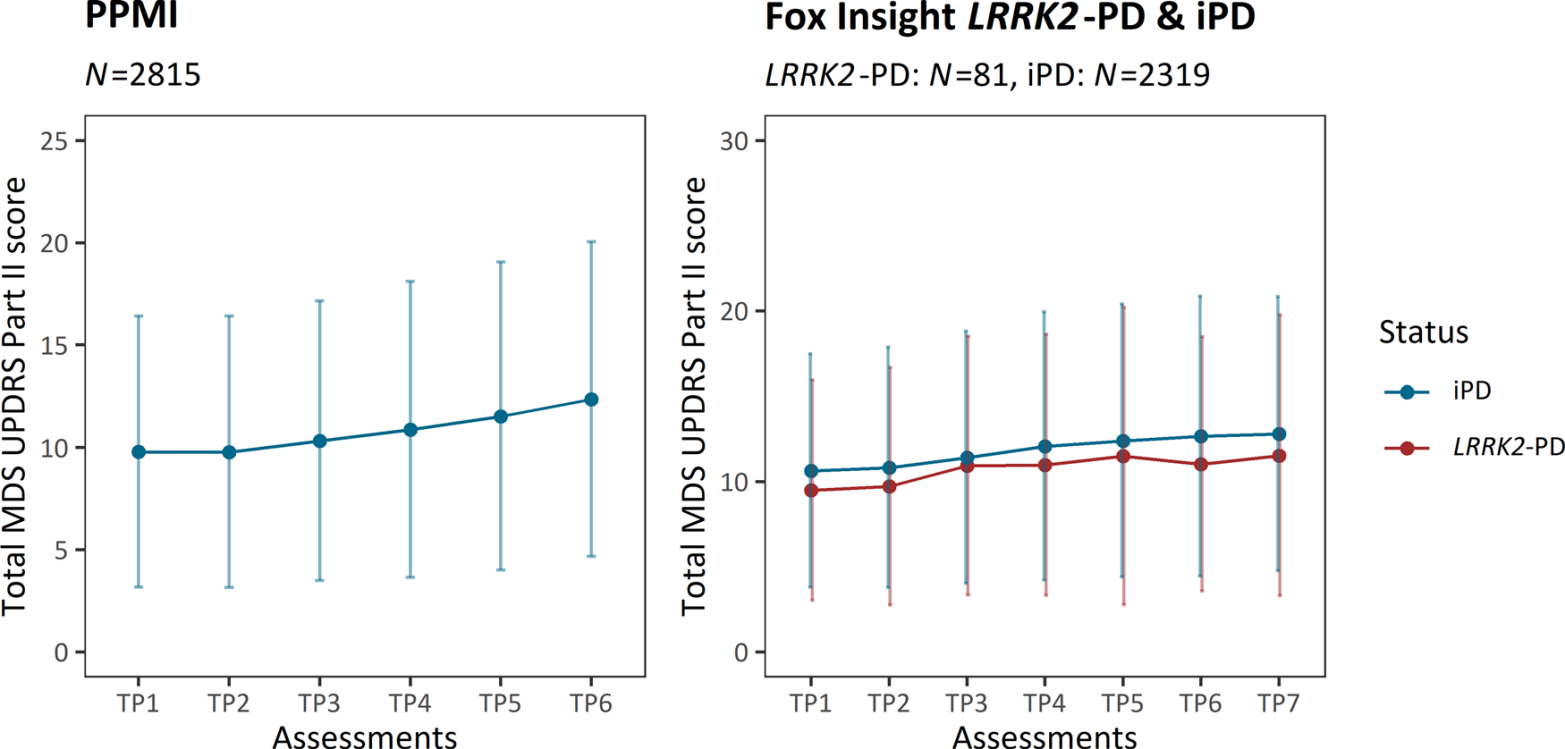
Motor aspect severity over time. The plots show the progression of PD motor features along the longitudinal assessments. At each time period, the mean cumulative MDS-UPDRS Part II score is indicated, and the error bars show the corresponding standard deviation. Patients with iPD are shown in blue (PPMI-Online and Fox Insight) and patients with *LRRK2*-PD are shown in red (Fox Insight). iPD Patients with idiopathic Parkinson’s disease, *LRRK2*-PD Patients with PD that carry the LRRK2 p.Gly2019Ser variant, TP Time period, *N* number of individuals.

We included patients with iPD from the PPMI-Online cohort. The mean enrollment time was 28.32 months, and the mean increase in motor features, as assessed with the MDS-UPDRS Part II, was 2.06 (Table 1). During the six assessment time periods, a gradual increase in motor aspect severity was observed (Fig. 2). The gradual rise of motor aspect severity was also illustrated with the linear mixed effects model, as there was an increasing effect size for each subsequent time period, demonstrating the longitudinal increase in motor severity (Table 2).

**Table 1:** Demographic overview of the longitudinal PD cohorts

	PPMI-Online	Fox Insight	
	iPD	iPD	*LRRK2*-PD
***N***	2815	2319	81
**Number of men (%)**	1690 (60.0%)	1247 (53.8%)	42 (51.9%)
**Mean AAE (** ± ***SD*)**	67.73 years ( ± 8.29)	65.95 years ( ± 8.12)	65.08 years ( ± 8.05)
**Mean AAO (** ± ***SD*)**	63.55 years ( ± 8.93)	61.57 years ( ± 8.83)	60.06 years ( ± 9.02)
**Mean disease duration at baseline (**±***SD*)**	4.18 years ( ± 4.08)	4.38 years ( ± 4.17)	5.52 years ( ± 4.53)
**Mean study enrollment time (**±***SD*)**	28.32 months ( ± 1.40)	52.54 months ( ± 2.52)	52.49 months ( ± 2.48)
**Mean motor aspects progression (**±***SD*)**	2.06 ( ± 4.81)	3.23 ( ± 6.14)	2.93 ( ± 5.38)

*N* Number of individuals, *AAE* Age at examination, *AAO* Age at onset, *iPD* Patients with idiopathic Parkinson’s disease, *LRRK2*-PD Patients with PD that carry the LRRK2 p.Gly2019Ser variant, *Motor aspects progression* (Cumulative MDS-UPDRS Part II score at the last assessment time period) - (Cumulative MDS-UPDRS Part II score at baseline).

**Table 2:** The motor aspect severity was evaluated longitudinally over time and assessed with a linear mixed model in the PPMI-Online and Fox Insight cohort

	Estimate	*SE*	*p*-value
**PPMI-Online (iPD:** ***N*** = **2815)**			
**Time period 2**	0.0001	0.01	0.993
**Time period 3**	0.07	0.01	9.78 × 10^−13^
**Time period 4**	0.12	0.01	<2.00 × 10^−16^
**Time period 5**	0.19	0.01	<2.00 × 10^−16^
**Time period 6**	0.26	0.01	<2.00 × 10^−16^
**AAO**	−0.05	0.01	1.57 × 10^−04^
**Disease duration**	0.64	0.03	<2.00 × 10^−16^
**Fox Insight (iPD:** ***N*** = **2319)**			
**Time period 2**	0.01	0.01	0.197
**Time period 3**	0.08	0.01	1.39 × 10^−11^
**Time period 4**	0.15	0.01	<2.00 × 10^−16^
**Time period 5**	0.21	0.01	<2.00 × 10^−16^
**Time period 6**	0.27	0.01	<2.00 × 10^−16^
**Time period 7**	0.31	0.01	<2.00 × 10^−16^
**AAO**	−0.03	0.01	0.019
**Disease duration**	0.71	0.05	<2.00 × 10^−16^
**OFF-episodes**	0.25	0.03	<2.00 × 10^−16^
**Fox Insight (*LRRK2*-PD:** ***N*** = **81)**			
**Time period 2**	−0.04	0.06	0.527
**Time period 3**	0.05	0.06	0.410
**Time period 4**	0.17	0.06	0.007
**Time period 5**	0.19	0.06	0.003
**Time period 6**	0.29	0.07	4.98 × 10^−5^
**Time period 7**	0.36	0.07	1.38 × 10^−7^
**AAO**	0.14	0.92	0.880
**Disease duration**	0.39	0.18	0.034
**OFF-episodes**	0.75	0.16	6.13 × 10^−6^
**Fox Insight (*LRRK2*-PD & iPD)**			
**Time period 2**	0.02	0.01	0.093
**Time period 3**	0.08	0.01	5.63 × 10^−14^
**Time period 4**	0.15	0.01	<2.00 × 10^−16^
**Time period 5**	0.22	0.01	<2.00 × 10^−16^
**Time period 6**	0.27	0.01	<2.00 × 10^−16^
**Time period 7**	0.32	0.01	<2.00 × 10^−16^
***LRRK2*-PD**	−0.23	0.08	0.005
**AAO**	−0.04	0.01	0.003
**Disease duration**	0.73	0.04	<2.00 × 10^−16^
**OFF-episodes**	0.27	0.03	<2.00 × 10^−16^

*N* Number of individuals, *iPD* Patients idiopathic Parkinson’s disease, *LRRK2-PD* Patients with PD that carry the LRRK2 p.Gly2019Ser variant, Formula in R (package lme4): lmer(log(Cumulative MDS-UPDRS Part II Score) ~ Assessment time periods+ mfp transformed AAO + mfp transformed disease duration + LRRK2-PD/iPD* + Experience of OFF episodes* + (1|Patient ID)), *If applicable.

Tested for significance at α = 0.017.

Baseline categories: Time period=Time period 1 (i.e., assessment at enrolment).

On the other hand, patients with iPD and *LRRK2*-PD were included from the Fox Insight cohort, where the patients had a mean enrollment time of 52.54 months and 52.49 months, respectively (Table 1). We observed more severe motor aspects over all seven assessments among patients with iPD (mean motor aspects progression=3.23) compared to patients with *LRRK2*-PD (mean motor aspects progression=2.93) (Table 1, Fig. 2). The less severe motor aspects of patients with *LRRK2*-PD were confirmed with a linear mixed effects model (β = −0.23, *SE* = 0.08, *p* = 0.005) (Table 2).

### Pesticide exposure in a work setting

**Fig. 3:**
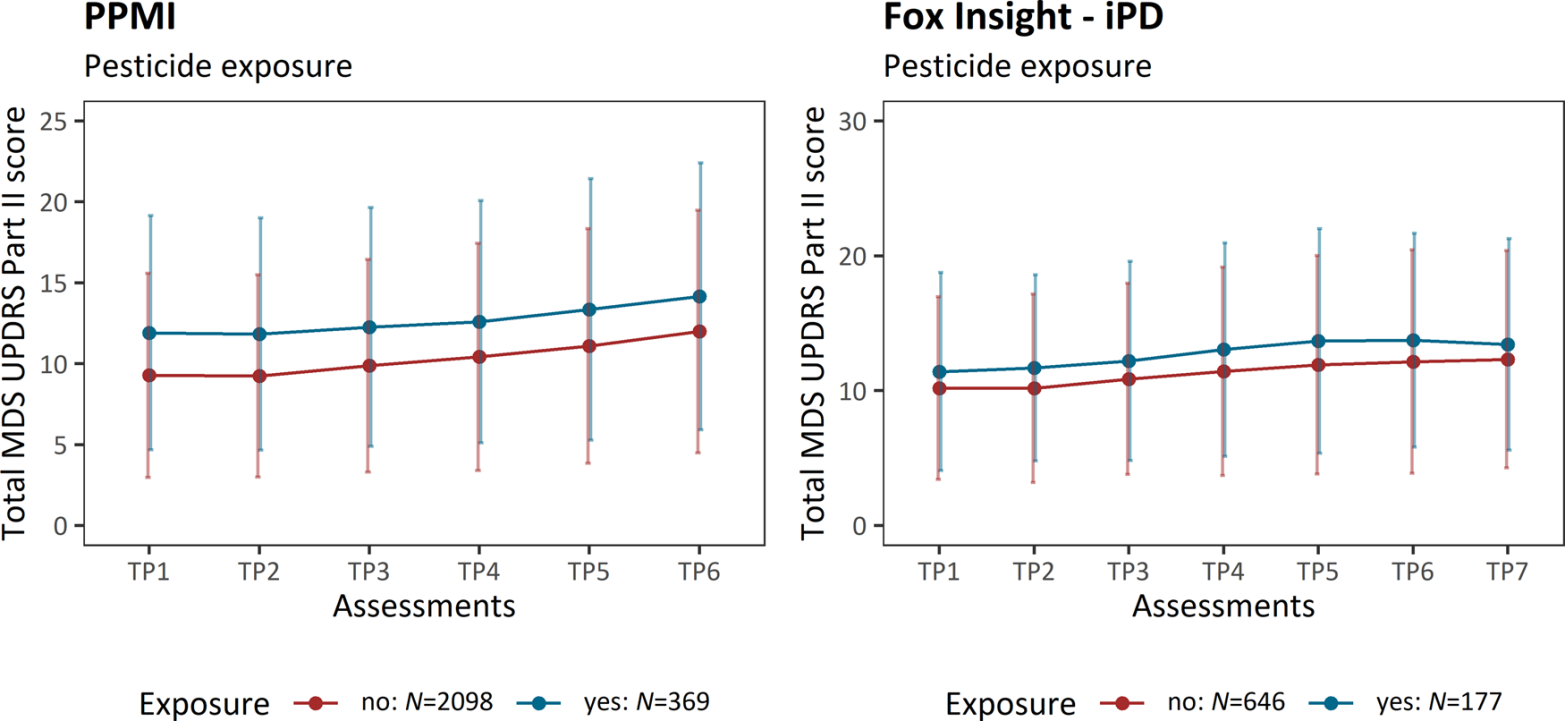
Motor aspect severity over time stratified by pesticide exposure. The plots show the progression of PD motor features along the longitudinal assessments. The mean cumulative MDS-UPDRS Part II score is indicated at each time period, and the error bars show the corresponding standard deviation. Patients with iPD are shown (PPMI-Online and Fox Insight). The patients are stratified by pesticide exposure. iPD idiopathic Parkinson’s disease, TP Time period, *N* number of individuals.

Within the PPMI-Online cohort, approximately 15% (*N* = 2467) of the patients with iPD were exposed to pesticides in a work setting. After the 35-month longitudinal assessment of motor aspects, iPD patients who were exposed to pesticides had more severe motor aspects (mean cumulative MDS-UPDRS Part II ( ± *SD*) = 14.15 ( ± 8.24)) compared to those who were not exposed (mean cumulative MDS-UPDRS Part II ( ± *SD*) = 11.99 ( ± 7.49)) (Fig. 3). The linear mixed effects model confirmed the significant association between pesticide exposure and motor severity, including six assessment time periods throughout the study (β = 0.23, *SE* = 0.04, *p* = 3.56 × 10^−9^, adjusted α = 0.017) (Table 3).

**Table 3:** The association between pesticide exposure and motor aspect severity over time

	Estimate	*SE*	*p*-value
**PPMI-Online (iPD: yes** **=** **369, no** = **2098)**			
**Time period 2**	0.001	0.01	0.890
**Time period 3**	0.07	0.01	4.18 × 10^−13^
**Time period 4**	0.12	0.01	<2.00 × 10^−16^
**Time period 5**	0.20	0.01	<2.00 × 10^−16^
**Time period 6**	0.27	0.01	<2.00 × 10^−16^
**Pesticide exposure**	0.23	0.04	3.56 × 10^−9^
**AAO**	−0.06	0.01	0.0002
**Disease duration**	0.63	0.04	<2.00 × 10^−16^
**Fox Insight (iPD: yes** = **177, no** = **646)**			
**Time period 2**	0.01	0.02	0.509
**Time period 3**	0.09	0.02	5.52 × 10^−6^
**Time period 4**	0.15	0.02	4.32 × 10^−14^
**Time period 5**	0.21	0.02	<2.00 × 10^−16^
**Time period 6**	0.27	0.02	<2.00 × 10^−16^
**Time period 7**	0.29	0.02	<2.00 × 10^−16^
**Pesticide exposure**	0.11	0.06	0.049
**AAO**	−0.02	0.02	0.459
**Disease duration**	0.77	0.09	<2.00 × 10^−16^
**OFF-episodes**	0.24	0.05	2.90 × 10^−7^
**Fox Insight (*LRRK2*-PD: yes** = **8, no** = **29)**			
**Time period 2**	−0.003	0.09	0.974
**Time period 3**	0.08	0.10	0.383
**Time period 4**	0.23	0.09	0.014
**Time period 5**	0.21	0.09	0.031
**Time period 6**	0.41	0.10	8.72 × 10^−5^
**Time period 7**	0.41	0.10	5.06 × 10^−5^
**Pesticide exposure**	−0.001	0.28	0.998
**AAO**	−2.01	1.56	0.207
**Disease duration**	0.36	0.29	0.227
**OFF-episodes**	0.55	0.24	0.030

*N* Number of individuals, *iPD* idiopathic Parkinson’s disease, *LRRK2*-PD Patients with PD that carry the LRRK2 p.Gly2019Ser variant, Formula in R (package lme4): lmer(log(Cumulative MDS-UPDRS Part II Score) ~ Assessment time periods + mfp transformed AAO + mfp transformed disease duration + Environmental/Lifestyle factor (yes/no) + Experience of OFF episodes* + (1|Patient ID))

*If applicable.

Baseline categories: Time period=Time period 1 (i.e., assessment at enrolment).

The motor aspect severity was evaluated longitudinally over time and assessed with a linear mixed model in the PPMI-Online and Fox Insight cohort.

In accordance with the data from the PPMI-Online cohort, we observed that iPD patients had visibly more severe motor aspects when they were exposed to pesticides (mean cumulative MDS-UPDRS Part II ( ± *SD*) = 13.42 ( ± 7.84)) compared to those that were not (mean cumulative MDS-UPDRS Part II ( ± *SD*) = 12.33 ( ± 8.04), Fig. 3). The association between pesticide exposure and increased motor aspect severity over time in iPD patients from Fox Insight was not significant after adjusting for multiple testing but the trend was in the same direction (β = 0.11, *SE* = 0.06, *p* = 0.049, Table 3). It is important to note that the linear mixed effects model was adjusted based on the experience of OFF episodes in the Fox Insight cohort but not in the PPMI-Online data, as this information was unavailable. The prevalence of pesticide exposure in a work setting was higher among iPD patients from Fox Insight ( ~ 22%, *N* = 823) compared to PPMI-Online.

In contrast, we did not observe an association between pesticide exposure and motor severity in patients with *LRRK2*-PD (β = −0.001, *SE* = 0.28, *p* = 0.998, *N* = 37).

### Smoking Behavior

**Fig. 4:**
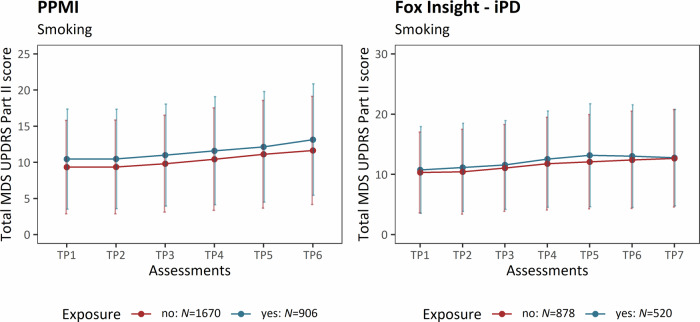
Motor aspect severity over time stratified by smoking. The plots show the progression of PD motor features along the longitudinal assessments. The mean cumulative MDS-UPDRS Part II score is indicated at each time period, and the error bars show the corresponding standard deviation. Patients with iPD are shown (PPMI-Online and Fox Insight). The patients are stratified by smoking status. iPD idiopathic Parkinson’s disease, TP Time period, *N* number of individuals.

The prevalence of smoking was ~35% among the patients with iPD in the PPMI-Online cohort (*N* = 2576). Interestingly, smoking status was associated with increased motor severity longitudinally (β = 0.13, *SE* = 0.03, *p* = 9.65 × 10^−6^) (Table 4). At the assessment time period six (i.e., 27–35 months), patients who smoked had more severe motor aspects (mean cumulative MDS-UPDRS Part II ( ± *SD*) = 13.14 ( ± 7.71)) compared to those who did not (mean cumulative MDS-UPDRS Part II ( ± *SD*) = 11.64 ( ± 7.48)) (Fig. 4).

**Table 4:** The association between smoking and motor aspect severity over time

	Estimate	*SE*	*p*-value
**PPMI-Online (iPD: yes** = **906, no** = **1670)**			
**Time period 2**	0.001	0.01	0.911
**Time period 3**	0.07	0.01	1.95 × 10^−11^
**Time period 4**	0.12	0.01	<2.00 × 10^−16^
**Time period 5**	0.20	0.01	<2.00 × 10^−16^
**Time period 6**	0.26	0.01	<2.00 × 10^−16^
**Smoking**	0.13	0.03	9.65 × 10^−6^
**AAO**	−0.05	0.01	0.001
**Disease duration**	0.63	0.04	<2.00 × 10^−16^
**Fox Insight (iPD: yes** = **520, no** = **878)**			
**Time period 2**	0.02	0.01	0.258
**Time period 3**	0.08	0.01	5.77 × 10^−9^
**Time period 4**	0.16	0.01	<2.00 × 10^−16^
**Time period 5**	0.23	0.02	<2.00 × 10^−16^
**Time period 6**	0.28	0.02	<2.00 × 10^−16^
**Time period 7**	0.31	0.02	<2.00 × 10^−16^
**Smoking**	0.06	0.04	0.122
**AAO**	−0.03	0.02	0.118
**Disease duration**	0.76	0.07	<2.00 × 10^−16^
**OFF-episodes**	0.24	0.04	4.19 × 10^−11^
**Fox Insight (*LRRK2*-PD: yes** = **22, no** = **28)**			
**Time period 2**	−0.02	0.07	0.831
**Time period 3**	0.05	0.08	0.493
**Time period 4**	0.20	0.08	0.008
**Time period 5**	0.16	0.08	0.035
**Time period 6**	0.34	0.08	5.30 × 10^−5^
**Time period 7**	0.37	0.08	1.02 × 10^−5^
**Smoking**	−0.07	0.20	0.735
**AAO**	−0.88	1.26	0.488
**Disease duration**	0.35	0.25	0.165
**OFF-episodes**	0.57	0.21	0.009

*N* Number of individuals, *iPD* idiopathic Parkinson’s disease, *LRRK2-PD* Patients with PD that carry the LRRK2 p.Gly2019Ser variant, Formula in R (package lme4): lmer(log(Cumulative MDS-UPDRS Part II Score) ~ Assessment time periods + mfp transformed AAO + mfp transformed disease duration + Environmental/Lifestyle factor (yes/no) + Experience of OFF episodes* + (1|Patient ID)).

*If applicable.

Baseline categories: Time period=Time period 1 (i.e., assessment at enrolment).

The motor aspect severity was evaluated longitudinally over time and assessed with a linear mixed model in the PPMI-Online and Fox Insight cohort.

We observed a subtle trend for increased motor aspect severity over time in smokers from the Fox Insight cohort, and this trend was limited to patients with iPD (β = 0.06, *SE* = 0.04, *p* = 0.122, *N* = 1398) and not present in *LRRK2*-PD (β = −0.07, *SE* = 0.20, *p* = 0.735, *N* = 50) (Table 4).

### Caffeinated beverage consumption

**Fig. 5:**
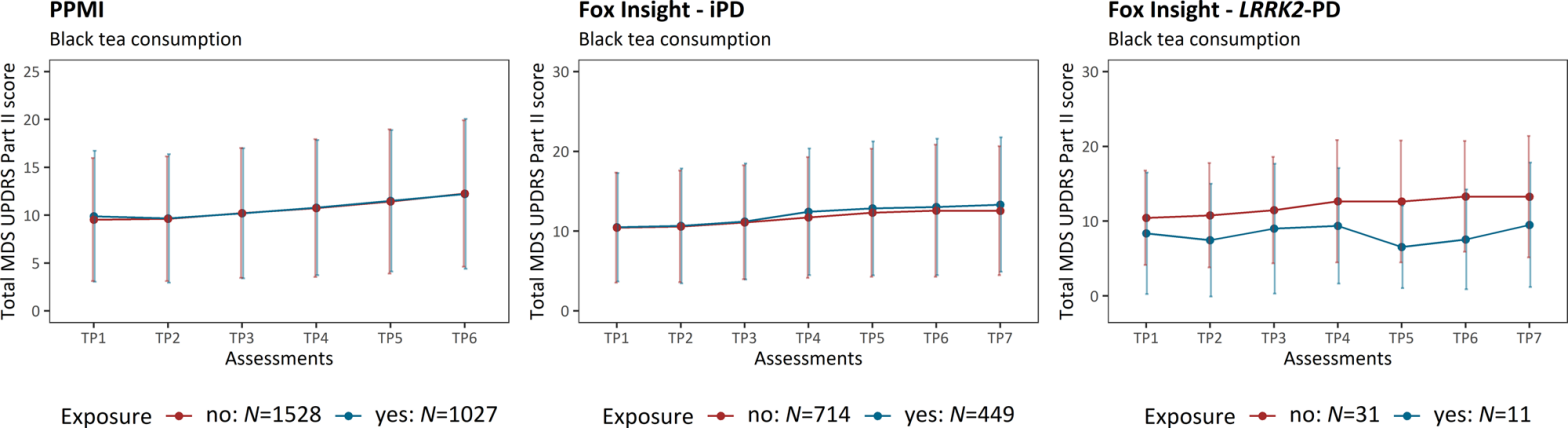
Motor aspect severity over time stratified by black tea consumption. The plots show the progression of PD motor features along the longitudinal assessments. The mean cumulative MDS-UPDRS Part II score is indicated at each time period, and the error bars show the corresponding standard deviation. Patients with iPD (PPMI-Online and Fox Insight) and *LRRK2*-PD (Fox Insight) are shown. The patients are stratified by black tea consumption. iPD idiopathic Parkinson’s disease, *LRRK2*-PD Patients with PD that carry the LRRK2 p.Gly2019Ser variant, TP Time period, *N* number of individuals.

Next, we investigated the longitudinal correlation between four caffeinated beverages (i.e., black tea, caffeinated soda, coffee, and green tea) and motor aspect severity. Black tea consumption prevalence was similar among the iPD patients of the PPMI-Online and Fox Insight cohort (PPMI-Online: ~40%, *N* = 2555; Fox Insight: 39%, *N* = 1213). However, we observed no difference in the motor severity over time when stratifying for black tea consumption (Fig. 5, Table 5).

**Table 5:** The association between black tea consumption and motor aspect severity over time

	Estimate	*SE*	*p*-value
**PPMI-Online (iPD: yes**= **1027, no** = **1528)**			
**Time period 2**	−0.004	0.01	0.715
**Time period 3**	0.07	0.01	8.03 × 10^−12^
**Time period 4**	0.12	0.01	<2.00 × 10^−16^
**Time period 5**	0.20	0.01	<2.00 × 10^−16^
**Time period 6**	0.26	0.01	<2.00 × 10^−16^
**Black tea**	0.005	0.03	0.872
**AAO**	−0.06	0.01	3.97 × 10^−5^
**Disease duration**	0.63	0.04	<2.00 × 10^−16^
**Fox Insight (iPD: yes** = **449, no** = **714)**			
**Time period 2**	0.01	0.02	0.534
**Time period 3**	0.08	0.02	1.6 × 10^−6^
**Time period 4**	0.15	0.02	<2.00 × 10^−16^
**Time period 5**	0.22	0.02	<2.00 × 10^−16^
**Time period 6**	0.28	0.02	<2.00 × 10^−16^
**Time period 7**	0.31	0.02	<2.00 × 10^−16^
**Black tea**	0.01	0.04	0.898
**AAO**	−0.02	0.02	0.186
**Disease duration**	0.68	0.07	<2.00 × 10^−16^
**OFF-episodes**	0.25	0.04	4.49 × 10^−10^
**Fox Insight (*LRRK2*-PD: yes** = **11, no** = **31)**			
**Time period 2**	−0.01	0.08	0.853
**Time period 3**	0.09	0.08	0.294
**Time period 4**	0.22	0.08	0.006
**Time period 5**	0.23	0.08	0.006
**Time period 6**	0.39	0.09	1.43 × 10^−5^
**Time period 7**	0.40	0.09	5.92 × 10^−6^
**Black tea**	−0.51	0.22	0.028
**AAO**	−1.01	1.26	0.427
**Disease duration**	0.38	0.24	0.123
**OFF-episodes**	0.57	0.21	0.009

*N* Number of individuals, iPD idiopathic Parkinson’s disease, *LRRK2-PD* Patients with PD that carry the LRRK2 p.Gly2019Ser variant, Formula in R (package lme4): lmer(log(Cumulative MDS-UPDRS Part II Score) ~ Assessment time periods + mfp transformed AAO + mfp transformed disease duration + Environmental/Lifestyle factor (yes/no) + Experience of OFF episodes* + (1|Patient ID)).

*If applicable.

Baseline categories: Time period=Time period 1 (i.e., assessment at enrolment).

The motor aspect severity was evaluated longitudinally over time and assessed with a linear mixed model in the PPMI-Online and Fox Insight cohort.

Interestingly, we specifically observed a large difference in motor aspect severity after 60-month enrollment between *LRRK2*-PD patients who consumed black tea (mean cumulative MDS-UPDRS Part II ( ± *SD*) = 9.50 ( ± 8.32)) and those who did not (mean cumulative MDS-UPDRS Part II ( ± *SD*) = 13.27 ( ± 8.11)). The linear mixed effects model confirmed the association between black tea and less severe motor aspects (β = −0.51, *SE* = 0.22, *p* = 0.028) (Table 5). Although the effect size of the association between black tea and motor aspects severity in *LRRK2*-PD was the largest out of all included environment/lifestyle factors, the sample size was small (*N* = 42, the prevalence of black tea consumption: ~26%), limiting the statistical power, potentially inflating the effect size, and requiring replication in a larger study cohort. Additionally, when including all iPD and *LRRK2*-PD patients from Fox Insight into one model and assessing the joint effect on motor severity of *LRRK2*-PD/iPD status and black tea consumption (i.e., LRRK2-PD/iPD status × Black tea consumption), there was evidence for an interaction effect between PD type (iPD or *LRRK2*-PD) and black tea consumption (β = −0.51, *SE* = 0.26, *p* = 0.045, *N* = 1333).

**Fig. 6:**
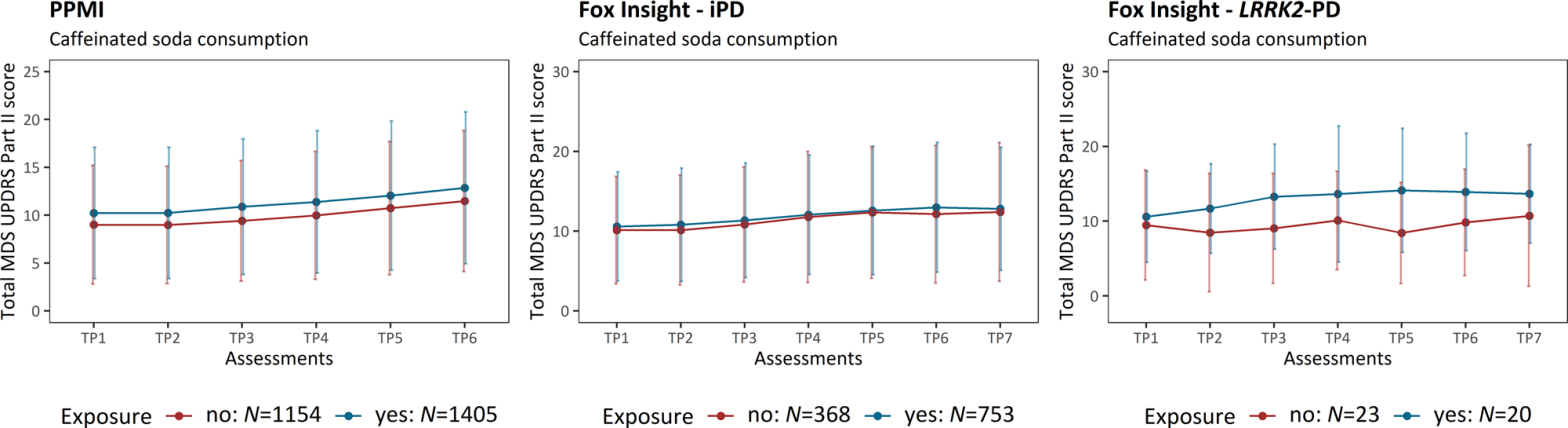
Motor aspect severity over time stratified by caffeinated soda consumption. The plots show the progression of PD motor features along the longitudinal assessments. The mean cumulative MDS-UPDRS Part II score is indicated at each time period, and the error bars show the corresponding standard deviation. Patients with iPD (PPMI-Online and Fox Insight) and *LRRK2*-PD (Fox Insight) are shown. The patients are stratified by caffeinated soda consumption. iPD idiopathic Parkinson’s disease, *LRRK2*-PD Patients with PD that carry the LRRK2 p.Gly2019Ser variant, TP Time period, *N* number of individuals.

In contrast to black tea consumption, caffeinated soda was associated with more severe motor aspects. The prevalence of caffeinated soda consumption was ~55% (*N* = 2559) in iPD patients of PPMI-Online. After the 35-month enrollment time, iPD patients who did drink caffeinated soda had more severe motor aspects (mean cumulative MDS-UPDRS Part II ( ± *SD*) = 12.85 ( ± 7.93)) compared to those who did not (mean cumulative MDS-UPDRS Part II ( ± *SD*) = 11.47 ( ± 7.37)) and the association was confirmed by the linear mixed model (β = 0.15, *SE* = 0.03, *p* = 3.84 × 10^−8^) (Fig. 6, Table 6).

**Table 6:** The association between caffeinated soda consumption and motor aspect severity over time

	Estimate	*SE*	*p*-value
**PPMI-Online (iPD: yes** = **1405, no** = **1154)**			
**Time period 2**	0.0005	0.01	0.964
**Time period 3**	0.07	0.01	1.18 × 10^−12^
**Time period 4**	0.12	0.01	<2.00 × 10^−16^
**Time period 5**	0.20	0.01	<2.00 × 10^−16^
**Time period 6**	0.26	0.01	<2.00 × 10^−16^
**Caffeinated soda**	0.15	0.03	3.84 × 10^−8^
**AAO**	−0.06	0.01	3.04 × 10^−5^
**Disease duration**	0.63	0.04	<2.00 × 10^−16^
**Fox Insight (iPD: yes** = **753, no** = **368)**			
**Time period 2**	0.01	0.02	0.533
**Time period 3**	0.08	0.02	5.48 × 10^−7^
**Time period 4**	0.15	0.02	<2.00 × 10^−16^
**Time period 5**	0.22	0.02	<2.00 × 10^−16^
**Time period 6**	0.28	0.02	<2.00 × 10^−16^
**Time period 7**	0.31	0.02	<2.00 × 10^−16^
**Caffeinated soda**	0.09	0.04	0.031
**AAO**	−0.02	0.02	0.189
**Disease duration**	0.72	0.07	<2.00 × 10^−16^
**OFF-episodes**	0.24	0.04	2.22 × 10^−9^
**Fox Insight (*LRRK2*-PD: yes** = **20, no** = **23)**			
**Time period 2**	−0.01	0.08	0.882
**Time period 3**	0.09	0.08	0.260
**Time period 4**	0.22	0.08	0.006
**Time period 5**	0.23	0.08	0.005
**Time period 6**	0.39	0.09	1.18 × 10^−5^
**Time period 7**	0.40	0.08	4.98 × 10^−6^
**Caffeinated soda**	0.35	0.21	0.108
**AAO**	−1.07	1.31	0.419
**Disease duration**	0.46	0.24	0.069
**OFF-episodes**	0.43	0.23	0.070

*N* Number of individuals, iPD idiopathic Parkinson’s disease, *LRRK2*-*PD* Patients with PD that carry the LRRK2 p.Gly2019Ser variant, Formula in R (package lme4): lmer(log(Cumulative MDS-UPDRS Part II Score) ~ Assessment time periods + mfp transformed AAO + mfp transformed disease duration + Environmental/Lifestyle factor (yes/no) + Experience of OFF episodes* + (1|Patient ID)).

*If applicable.

Baseline categories: Time period=Time period 1 (i.e., assessment at enrolment).

The motor aspect severity was evaluated longitudinally over time and assessed with a linear mixed model in the PPMI-Online and Fox Insight cohort.

In patients with iPD from the Fox Insight cohort, the prevalence of caffeinated soda consumption was ~67% (*N* = 1121). After an enrollment time of 60 months, the iPD patients from Fox Insight who consumed caffeinated soda had more severe motor aspects (mean cumulative MDS-UPDRS Part II ( ± *SD*) = 12.79 ( ± 7.72)) compared to those who did not as well (mean cumulative MDS-UPDRS Part II ( ± *SD*) = 12.41 ( ± 8.70)) and the association was confirmed by the linear mixed model (β = 0.09, *SE* = 0.04, *p* = 0.031). With regard to the *LRRK2*-PD patients (prevalence of caffeinated soda consumption: ~47%, *N* = 43), patients who consumed caffeinated soda had visibly more severe motor aspects (mean cumulative MDS-UPDRS Part II ( ± *SD*) = 13.67 ( ± 6.61)) compared to the *LRRK2*-PD patients that did not (mean cumulative MDS-UPDRS Part II ( ± *SD*) = 10.71 ( ± 9.43)) (Fig. 6). However, the association was not confirmed when the linear mixed model was applied (β = 0.35, *SE* = 0.21, *p* = 0.108) (Table 6).

Coffee and green tea consumption were explored analogously to the other caffeinated beverages; however, we did not observe an association with motor severity over time in patients with iPD or *LRRK2*-PD (Supplementary Tables 1, 2).

### Association between sex, environment, lifestyle and motor aspects severity

It is known that the prevalence of particular environmental exposures and lifestyle factors can be different between women and men^26^. We also observed that the prevalence of pesticide exposure was visibly higher in men, and that of caffeinated soda and smoking was subtly higher in men. On the other hand, black tea has a more balanced prevalence among men and women or even a higher prevalence among women (Supplementary Fig. 1). Thus, we adjusted the mixed linear models for sex and evaluated if the associations we observed above remained. Analogously, we assessed all other environmental and lifestyle factors without an observed association with motor aspects, adjusting the models for sex, but all other analyses remained unchanged.

Pesticide exposure was associated with more severe motor aspects in patients with iPD in the PPMI-Online cohort. When adjusting for sex, the linear mixed effects model confirmed the significant association between pesticide exposure and motor severity in PPMI-Online (β = 0.19, *SE* = 0.04, *p* = 1.24 × 10^−6^, adjusted α = 0.017, Supplementary Table 3A), and there still was a trend in the same direction when assessing iPD patients from Fox Insight (β = 0.08, *SE* = 0.06, *p* = 0.146).

Secondly, we detected an association between smoking and motor aspects across time in patients with iPD in PPMI-Online, which remained unchanged after adjusting for sex (β = 0.12, *SE* = 0.04, *p* = 3.17 × 10^−5^, Supplementary Table 3B).

Thirdly, we detected an association between black tea consumption and less severe motor aspects across time in patients with *LRRK2*-PD, specifically, which also remained unchanged after adjusting for sex (β = −0.53, *SE* = 0.23, *p* = 0.028, Supplementary Table 3C).

Lastly, with regard to caffeinated soda consumption, we detected an association with more severe motor aspects in iPD (PPMI-Online and Fox Insight) and a similar trend in *LRRK2*-PD. The association with iPD remained after adjusting for sex in PPMI-Online (β = 0.13, *SE* = 0.03, *p* = 2.60 × 10^−5^, Supplementary Table 3D) but got weaker in Fox Insight (iPD: β = 0.07, SE = 0.04, *p* = 0.119, *LRRK2*-PD: β = 0.35, *SE* = 0.22, *p* = 0.111). The large sample size of men and women consuming caffeinated soda within PPMI-Online allowed us to stratify the cohort of iPD patients by sex and investigate the association in men and women individually. It is important to note that there was a much stronger association between caffeinated soda and motor aspect severity over time in female iPD patients (β = 0.23, *SE* = 0.05, *p* = 5.0 × 10^−7^) compared to male (β = 0.06, *SE* = 0.03, *p* = 0.062).

## Discussion

Investigating the impact of environmental and lifestyle factors beyond the onset of PD is of great importance, as there is increasing evidence that those factors affect the disease course. Thus far, there has been no longitudinal study on the association between smoking, caffeine consumption, and PD features severity. Therefore, we utilized large available data sets with longitudinal follow-ups of patients with PD (i.e., PPMI-Online and Fox Insight) to thoroughly investigate the effect of pesticide exposure, smoking, and caffeine over time. Additionally, we explored the association between environment, lifestyle, and motor severity in patients with monogenic *LRRK2*-PD in comparison to iPD. Longitudinal cohorts have major advantages over studies with only one study time point, as exposures are recorded at an early study stage (e.g., Fox Insight at study enrollment), resulting in a higher potential of a causal relationship. In the case of PPMI-Online, the assessment of environmental/lifestyle factors was not done at the study enrollment but at different time points throughout the study. However, we utilized the assessment of PPMI-Online, in which the largest number of participants answered the respective environmental and lifestyle questionnaires, to maximize the sample size and statistical power.

The progression in the MDS-UPDRS part II score during the longitudinal assessment was in the expected range^34^, but patients with *LRRK2*-PD had less severe motor aspects than patients with iPD. This finding concords with a recent 3.5-year longitudinal study demonstrating less severe PD features in patients with *LRRK2*-PD^5^. They found that patients with iPD and *LRRK2*-PD differed the most in their non-motor PD features, and *LRRK2*-PD patients presented with predominantly motor impairment. Here, we could show that patients with *LRRK2*-PD consistently present with less severe motor features over the complete enrollment time as well (Fig. 2).

Pesticide exposure is the most substantial environmental risk factor for PD^6^ and is associated with an earlier disease onset as well^8,9^. In line with increased risk and earlier AAO, we found that pesticide exposure was significantly associated with increased motor aspects in iPD, thus further underlying the link between pesticides and PD pathogenesis. Our study adds to the increasing evidence that pesticide exposure impacts patients with PD beyond the onset of the disease, as another longitudinal study recently found specific pesticides to be associated with faster PD progression^11^.

Our study demonstrated the association between occupational pesticide exposure and motor aspects in the large PPMI-Online cohort. Although the association between exposure and more severe motor aspects did not reach significance in the Fox Insight cohort, the trend was in the same direction and iPD patients that were exposed to pesticides had a visible higher motor aspect severity over time as well. However, we did not detect an association in *LRRK2*-PD; thus, they might not be as vulnerable to pesticide exposure. Although our sample size of *LRRK2*-PD patients with available pesticide information was limited and therefore, the statistical power was low, our group demonstrated before that non-occupational pesticide exposure was explicitly associated with a seven-year earlier median AAO in patients with iPD but not in *LRRK2*-PD in an independent cohort^26^. Recently, a larger-sized study also found no association between LRRK2 G2019S penetrance and pesticide exposure^10^, which further questions the importance of pesticide exposure as an *LRRK2*-PD modifier. The same study found that there was an increased disease risk among carriers of PD-related *GBA1* variants^10^. Classically, pesticides like rotenone and paraquat are known to impair mitochondrial function^35^, and there is increasing evidence that lysosomal and autophagy dysfunction has been shown to increase PD susceptibility in persons with heavy pesticide exposure^36^. It is important to note that lysosomal autophagic dysfunction is one of the key players in the pathogenesis of *GBA1*-related PD^37–39^, which might explain the increased PD risk in *GBA1* variant carriers. Although lysosomal impairment has also been observed in *LRRK2*-related PD^40–42^, the pathogenesis of *LRRK2*-PD is much more diverse and less well elucidated, including mitochondrial dysfunction, vesicle trafficking, and protein scaffolding among other pathways^43–45^.

The harmful impact of pesticide exposure, even beyond the onset of PD, is particularly important to highlight. In contrast to unfavorable lifestyle factors, which can be optimized in patients or persons at risk, occupational or residential pesticide exposure is much harder to avoid, and exposure can happen unconsciously. In our longitudinal study, we focused on occupational pesticide exposure, as the data was available in both assessed longitudinal cohorts. Occupational exposure is less prevalent in the population than residential exposure. Additionally, recall biases might impact self-reported exposure, which applies to all self-reported environmental or lifestyle data.

Smoking is one of the lifestyle factors with the most considerable protective effect on PD^6^, and is associated with a later AAO in iPD and *LRRK2*-PD^22,23,29^, and the protective effect might even be causal^46^. In contrast, smoking was associated with more severe motor aspects over time in our study. The association was detected in iPD patients from PPMI-Online, but there was also a visible trend for increased motor severity in iPD patients from Fox Insight. Compared to pesticide exposure, the effect size was smaller and the association was only present in one of the cohorts. Therefore, smoking might not be as robustly associated with more severe motor aspects. Nevertheless, the results of our longitudinal study support the findings from a cross-sectional assessment of smoking and increased disease severity^28^. There is evidence that the protective effect of smoking might be restricted to the time window before the disease onset. Indeed, smoking is associated with increased all-cause mortality and various health consequences, such as different forms of cancer, aortic aneurysms, peripheral artery diseases and chronic obstructive pulmonary disease^47–49^. Therefore, it may be the case that the overall harmfulness of smoking overshadows the PD-specific protective effects after disease onset.

The neuroprotective effect of smoking is most likely mediated by nicotine and its stimulating effects on dopaminergic neurons via neuronal nicotinic acetylcholine receptors (nAChRs)^50^. Interestingly, it has recently been shown that the time window for the beneficial neuroprotective effects of nicotine is very small and likely ineffective after the majority of dopaminergic neurons are lost^51,52^. Similarly, a double-blinded phase-II trial evaluating the therapeutic effect of nicotine on motor signs and symptoms in PD patients showed no benefits of nicotine. There was even a trend for a more substantial progression in the nicotine-treated group compared to the placebo group^53^. There is a substantial reduction of nAChRs that even precedes the dopaminergic neuron loss in PD patients^50^. Thus, it is plausible that due to the narrow therapeutic window of nicotine, patients who already show motor symptoms do not benefit from the neuroprotective effect anymore^51^.

Lastly, we assessed caffeine consumption, including coffee, black tea, green tea, and caffeinated soda. Coffee and tea are also considered protective lifestyle factors in the context of PD^6^ and our group previously reported a strong correlation between black tea consumption and later AAO in patients with *LRRK2*-PD^26^. We did not observe any association between coffee or green tea and motor severity over time in our study cohorts. However, black tea was associated explicitly with less severe motor aspects in *LRRK2*-PD, and there was no difference in patients with iPD stratified for black tea consumption. As the sample size of longitudinally followed-up patients with *LRRK2*-PD and available lifestyle data in the Fox Insight cohort was limited, we investigated the association between motor aspects and black tea consumption in an independent longitudinal cohort. Therefore, we utilized the PPMI cohort^33^ and only participants not enrolled in the PPMI-Online sub-study (*LRRK2*-PD: *N* = 39, enrollment time = 90 months). Interestingly, we could confirm the association between black tea consumption and less severe motor aspects in patients with *LRRK2*-PD (β = −0.41, *SE* = 0.18, *p* = 0.026) (Supplementary Fig. 2, Supplementary Table 4). It is important to note that black tea consumption was the only factor in our study associated with decreased motor severity over time, and the association was robustly present in two independent *LRRK2*-PD cohorts. Subsequently, the protective effect of black tea consumption might remain present after disease onset. The underlying molecular pathway of the potential protective effect of black tea consumption is not fully understood. There is evidence that specific tea components have a neuroprotective effect^54^ and prevent apoptosis of dopaminergic neurons by increasing mitochondrial biogenesis^55^. Indeed, mitochondrial dysfunction is essential for PD pathogenesis; still, it might not affect all patients with PD. Thus, black tea consumption potentially has a larger effect on *LRRK2*-PD patients, who experience more likely mitochondrial dysfunction compared to the inhomogeneous group of patients with iPD. On the other hand, caffeine can act as a non-selective adenosine A2 receptor antagonist, blocking adenosine receptors and protecting against neurodegeneration and modulating dopaminergic pathways^52,56–58^. It has been proposed that in the presence of the pathogenic LRRK2 G2019S variant, the neuroprotective effect of caffeine via adenosine A2 receptor might be amplified due to the increase in LRRK2 kinase activity^59^. The reason why we observe this negative association exclusively with black tea consumption rather than other caffeinated beverages remains unclear. Future functional studies will be necessary to elucidate the precise molecular mechanism underlying this phenomenon.

In contrast to black tea, we found that caffeinated soda consumption was consistently associated with more severe motor aspects in patients with iPD from PPMI-Online and Fox Insight, underlining the association’s robustness. In addition, there was also a strong visible trend for more severe motor aspects in *LRRK2*-PD patients who consumed caffeinated soda, but the association did not reach significance. Our group has previously shown that caffeinated soda, different from other caffeinated beverages, is not a protective factor in PD and is associated with earlier disease onset in patients with *LRRK2*-PD^26^ and might increase the vulnerability of *LRRK2*-PD patients for increased mitochondrial dysfunctions^29^. Thus far, the underlying molecular pathway mediating the effects of caffeinated soda is unknown. However, as coffee and tea are protective lifestyle factors in PD, caffeine-independent mechanisms might be involved.

Comparing black tea and caffeinated sodas and their opposite effect on motor severity over time, sodas probably differ from tea in terms of ingredients and higher sugar content. In the last years, type 2 diabetes (T2DM) has been elucidated as an important comorbidity in the context of PD^60^. T2DM has even been associated with more severe motor signs^61^, and GLP1 receptor agonists, which are T2DM treatments, have recently been found to hold the progression of motor signs in PD patients^62^. Certainly, particular lifestyle and dietary behaviors can also increase the risk of developing T2DM. Unfortunately, the sample size of patients with PD and T2DM in our study cohorts was too small to explore the joint effects of T2DM and different lifestyle factors (Supplementary Table 5). Still, potential interactions should be considered for future studies. The underlying pathway of how caffeinated soda or black tea potentially affect motor severity has not been elucidated yet. The high sugar content of regular caffeinated sodas might be a potential driver of the observed association. Thus, we explored the consumption of caffeinated diet soda as well. Information on this beverage group was only available within PPMI-Online (prevalence of caffeinated diet soda consumption in PPMI-Online: ~43%, *N* = 2556). Interestingly, we did not observe an association with motor aspect severity over time (Supplementary Fig. 3). This finding highlights the importance of exploring high sugar intake as a driver of the observed association between regular caffeinated soda and motor severity. Unfavorable diet behaviors, including high sugar intake, might also affect the gut microbiome, and future studies should investigate a potential link to a faster PD progression. However, our study provides evidence that certain caffeinated beverages are longitudinally associated with motor aspect severity and may influence PD patients beyond disease onset. In light of the opposite direction of the effects of black tea and caffeinated soda, we explored if there are any joint associations; however, we did not observe joint independent or interactions between black tea and caffeinated soda on motor aspect severity. Furthermore, there is no correlation between the two beverages, as a substantial fraction of participants consumed both beverages, only one or none (Supplementary Fig. 4).

Lastly, we observed that male iPD patients had more severe motor symptoms over time (Supplementary Table 3A–D). This finding aligns with the literature, as there is evidence that women present with a more benign phenotype at the beginning of the disease^63^. In contrast, we did not observe an association between male sex and motor aspects in *LRRK2*-PD. Although the prevalence of certain environmental and lifestyle factors varied enormously between men and women, our analysis remained unchanged for the most part when adjusting for sex. However, the association between caffeinated soda consumption and more severe motor symptoms weakened after adjusting for sex as a covariate. This attenuation can be attributed to the higher prevalence of male patients among those consuming caffeinated soda, as male sex itself was associated with greater motor symptom severity over time. Moreover, a stronger association between caffeinated soda consumption and motor symptoms was observed in women compared to men, suggesting a potential sex-specific effect. Thus, our study contributes to the growing body of evidence supporting the sex-dependent effects of caffeinated beverages on Parkinson’s disease clinical severity and risk^64,65^. Within our study, we utilized the MDS UPDRD Part II (i.e., motor aspects of experiences of daily living), as the data source were the online cohorts Fox Insight and PPMI-Online, containing self-reported participant data. As a future perspective, exploring the (sex-specific) associations of environmental and lifestyle factors with the MDS UPDRD Part III (i.e., motor examination) would be of importance.

The main strength of our study is the large sample size of patients with iPD who were consistently followed up over a long period of time. Evaluating the association between environment, lifestyle and motor severity over time at consecutive points in time allows a more comprehensive assessment compared to a cross-sectional one. Investigating the environment and lifestyle in two cohorts demonstrates the reproducibility and robustness of our observed associations. The availability of genetic data for two PD subtypes in Fox Insight made it possible to distinguish between iPD and *LRRK2*-PD, which is essential as certain environmental or lifestyle factors may affect patients with other PD subtypes differently. Most participants in the study cohorts were patients with iPD, resulting in a small sample size of *LRRK2*-PD patients to explore, which is one of the largest limiting factors of our study. Thus, these findings concerning *LRRK2*-PD are fully exploratory and larger cohorts are required for replication. Additionally, our study focuses on patients with iPD and *LRRK2*-PD, neglecting other monogenic forms of PD. Given our findings, we recognize the importance of investigating additional PD subtypes. There were *N* = 162 patients with *GBA1*-PD and longitudinal data. However, *GBA1*-PD had comparable motor aspect severity over time to patients with iPD (β = −0.03, *SE* = 0.05, *p* = 0.437). We did not detect a clear association with any of the evaluated environmental/lifestyle factors and motor aspects in *GBA1*-PD. Genes strongly linked to mitochondrial dysfunction, such as *PRKN*-PD, *DJ-1*-PD, and *PINK1*-PD, warrant future investigation. As these variant carriers are even rarer, addressing this research question in the future would require larger cohorts with a specific focus on recruiting patients with these monogenic forms of PD. Although the sample sizes of patients with monogenic forms remain small in most of the research cohorts, we strongly recommend analyzing different PD subtypes separately when assessing environmental and lifestyle factors, as our findings and those of others suggest these subtypes exhibit distinct patterns.

Additionally, the PD medication dose can be another confounder that should be adjusted for; however, variables such as levodopa equivalent dose were unavailable from both research cohorts. Other potential confounding factors, particularly concerning lifestyle factors, are certain dietary behaviors such as sugar or dessert intake frequency as well as risk-seeking (e.g., risk-seeking gambling, drug abuse or extreme sports). Unfortunately, that information is unavailable in the PPMI-Online study and present only for a small subset of patients in the Fox Insight study, limiting our possibility of adjusting for these factors.

Another limitation is that recall bias can impact the assessment of environmental and lifestyle factors, as included in this study. In addition, pesticide exposure can even happen without the participants’ knowledge. More objective ways would be utilizing geographical databases of pesticide usage or personal sensor measuring devices, which can benefit an accurate assessment of the environment and lifestyle. Larger studies, including sufficient sample sizes of other monogenic PD forms, will also be required to elucidate and differentiate the impact of environmental factors on different PD subtypes and their disease progression.

The results of our longitudinal study provide further evidence of the importance of environment and lifestyle in PD, even after the disease onset. We observed that pesticide exposure and caffeinated soda are associated with more severe motor aspects in iPD. Additionally, smoking might also be associated with motor severity but with a smaller effect size. In patients with *LRRK2*-PD, primarily black tea consumption was associated with less severe motor aspects, and the protective effect was specific to *LRRK2*-PD and not present in iPD. Thus, environmental exposure and lifestyle factors affect motor severity in patients with *LRRK2*-PD and iPD. Nevertheless, further replication with larger sample sizes of monogenic PD will be required alongside functional studies elucidating the underlying molecular pathways of how motor severity is impacted by specific environmental exposures or lifestyle factors.

## Methods

### Longitudinal cohorts and participants demographics

The online cohorts Fox Insight and PPMI-Online are focused on PD research, and both provide a platform for patients to participate. The Fox Insight data consists of up to five years of routine longitudinal assessments (health and medical questionnaires) and additional one-time questionnaires about, e.g., environmental exposure and lifestyle habits assessed at enrollment^31^. The PPMI-Online study is also an online cohort, recruiting participants with and without PD. It is linked to the PPMI study and includes longitudinal assessments of motor and non-motor features of PD and environmental/lifestyle data assessed at different study time points^32,33^.

The Fox Insight and PPMI cohorts are established data resources from the Michael J. Fox Foundation. Participants provide informed consent through the Fox Insight website; informed consent and study protocol are reviewed by the WCG IRB (IRB#: 120160179, Legacy IRB#: 14–236, Sponsor Protocol Number: 1, Study Title: Fox Insight)^31^. The PPMI study was conducted in accordance with the Declaration of Helsinki and the Good Clinical Practice (GCP) guidelines after approval of the local ethics committees of the participating sites^32,33^. Ethical approval was obtained from the Ethics Committee of the University of Lübeck.

The participants were filtered for AAO > 25 years, disease duration <50 years and at least three longitudinal assessments of motor aspects. This data harmonization step was included to ensure an unbiased approach without extreme outliers for a fair outcome of the statistical analysis and to obtain an approximately equal number of patients per assessment. We included *N* = 2815 patients with iPD from the PPMI-Online cohort and *N* = 2319 from Fox Insight. In addition, we included *N* = 81 patients with *LRRK2*-PD from Fox Insight (Fig. 1). In this study, we focused on PD motor features as those were assessed with the same questionnaire in both cohorts, and it has been reported previously that *LRRK2*-PD patients are predominantly affected by motor impairments^5^.

In patients with iPD, there was a similar AAO (PPMI-Online: mean AAO ( ± *SD*) = 63.55 years ( ± 8.93), Fox Insight: mean AAO ( ± *SD*) = 61.57 years ( ± 8.83)) and disease duration (PPMI-Online: mean disease duration ( ± *SD*) = 4.18 years ( ± 4.08), Fox Insight: mean disease duration ( ± *SD*) = 4.38 years ( ± 4.17)) at baseline in both cohorts. However, patients with *LRRK2*-PD had a slightly younger AAO (mean AAO ( ± *SD*) = 60.06 years ( ± 9.02)) and longer disease duration (mean disease duration ( ± *SD*) = 5.52 years ( ± 4.53)) (Table 1). The severity of motor aspects was assessed using the MDS-UPDRS Part II score (i.e., Motor Aspects of Experiences of Daily Living). The individual scores for PD motor features included in the questionnaire were added to obtain the cumulative MDS-UPDRS Part II score.

Participants of the PPMI-Online cohort were followed up for up to 35 months (mean study enrollment time ( ± *SD*): 28.32 months ( ± 1.40)) and participants of the Fox Insight cohort for up to 60 months (iPD: mean study enrollment time ( ± *SD*) = 52.54 months ( ± 2.52), *LRRK2*-PD: mean study enrollment time ( ± *SD*) = 52.49 months ( ± 2.48)). By calculating the months since the baseline (at 0 months) of the individual assessments, we assigned them to one of either six or seven different assessment time periods in the PPMI-Online or Fox Insight data sets, respectively. The 35 month enrollment study time of the PPMI-Online cohort was divided into six to nine-month-long time periods, depending on the number of observations per time period (Supplementary Fig. 5A). The 60-month enrollment study time of the Fox Insight cohort was divided into 10-month-long time periods (Supplementary Fig. 5B, C). In the rare case that a patient was assessed two times per assigned time period, we included only the first assessment in our analysis.

### Environmental and lifestyle factors

The PD Risk Factor Questionnaire (PD RFQ-U) was used to assess environmental and lifestyle information in the PPMI-Online and Fox Insight cohort. The PD RFQ-U questionnaires are available under the ‘Resources’ section on the Fox Insight website. (https://foxden.michaeljfox.org/insight/explore/insight.jsp). In our study, we utilized the caffeine, tobacco and pesticide exposure at work parts of the PD RFQ-U. In the PD RFQ-U, pesticide exposure in a work setting is defined as being ever exposed to pesticides in your job (PD RFQ-U question: Over your lifetime, have you ever had a JOB in which you mixed, applied, or were exposed in some other way to any type of pesticide, including herbicides (kill weeds), fungicides (kill fungus/mold), insecticides (kill insects), rodenticides (kill rats/mice), or fumigants (gas used to kill fungus/mold or insects)?). Smoking is defined as smoking more than 100 cigarettes in your lifetime (PD RFQ-U question: In your lifetime, have you smoked 100 or more cigarettes?). Caffeine consumption is defined as drinking coffee, black tea, green tea or caffeinated soda at least once per week for more than six months. (PD RFQ-U question: In your lifetime, have you ever regularly drunk caffeinated [beverage name], that is, at least once per week for 6 months or longer?).

### Linear mixed effects model

To evaluate the longitudinal data statistically, we applied linear mixed effects models. The model’s outcome was the log-transformed cumulative MDS-UPDRS Part II score (range 0–52) to achieve a more appropriate normal distribution of the outcome variable (Supplementary Fig. 6). We included time (six or seven time periods, categorical variable), disease duration (in years), AAO (in years), and experience of OFF episodes (binary yes/no variable) as fixed effects and the patient ID as the random effect in the model. The continuous fixed effect covariates (i.e., AAO & disease duration) were transformed using fractional polynomials to achieve a more linear correlation with the outcome variable (Supplementary Fig. 7). The information about the experience of OFF episodes was only available for the Fox Insight cohort (Fig. 1). In Fox Insight, we also applied the mixed linear effect model to the complete data set, including patients with iPD together with *LRRK2*-PD patients into one model and the *LRRK2*-PD/iPD status as a binary variable as an additional fixed effect.

Linear mixed effects models were also applied to explore the association between environmental exposures, lifestyle factors and motor severity over time. The individual environmental and lifestyle factors were included as fixed effects in the form of binary variables (yes/no). In an additional analysis, we investigated the potential association between biological sex (binary variable), environment/lifestyle and motor aspects.

The analyses were performed in R (v.4.3.1)^66^ with the *lme4* (v.1.1-34)and *lmerTest* (v.3.1-3) packages^67,68^. The continuous fixed-effect covariates were transformed with a multiple fractional polynomials (*mfp*) function from the *mfp* (v. 1.5.4) package^69^. The analysis of the association between pesticide exposure and motor aspect severity was interpreted for significance, based on the presence of the ‘a priori’ hypothesis on the association between pesticide and PD severity. The association with pesticides was tested for significance in iPD patients from PPMI-Online, Fox Insight and *LRRK2*-PD patients from Fox Insight. Thus, the significance level was adjusted for performing three tests to α = 0.05/3 = 0.017. All other analyses in this study were exploratory and *p*-values were not corrected for multiple testing.

## Supplementary information

Supplementary Material

## Data Availability

Data used in the preparation of this article were obtained from the Fox Insight database (https://foxinsight-info.michaeljfox.org/insight/explore/insight.jsp) on 24/07/2024. For up-to-date information on the study, visit https://foxinsight-info.michaeljfox.org/insight/explore/insight.jsp. Data used in the preparation of this article were obtained 24/07/2024 from the Parkinson’s Progression Markers Initiative (PPMI) database (www.ppmi-info.org/access-dataspecimens/download-data), RRID:SCR 006431. For up-to-date information on the study, visit www.ppmi-info.org.
