# Supplementary material for: Longitudinal assessment of the association between pesticide exposure and lifestyle with Parkinson’s disease motor severity
Source: NPJ Parkinsons Dis. 2025 Jun 12;11:164. doi: 10.1038/s41531-025-01010-2 (PMC12162858; doi:10.1038/s41531-025-01010-2)
Supplement: Supplementary file 1 — Supplementary Material [file 41531_2025_1010_MOESM1_ESM.pdf]

Supplementary Material:

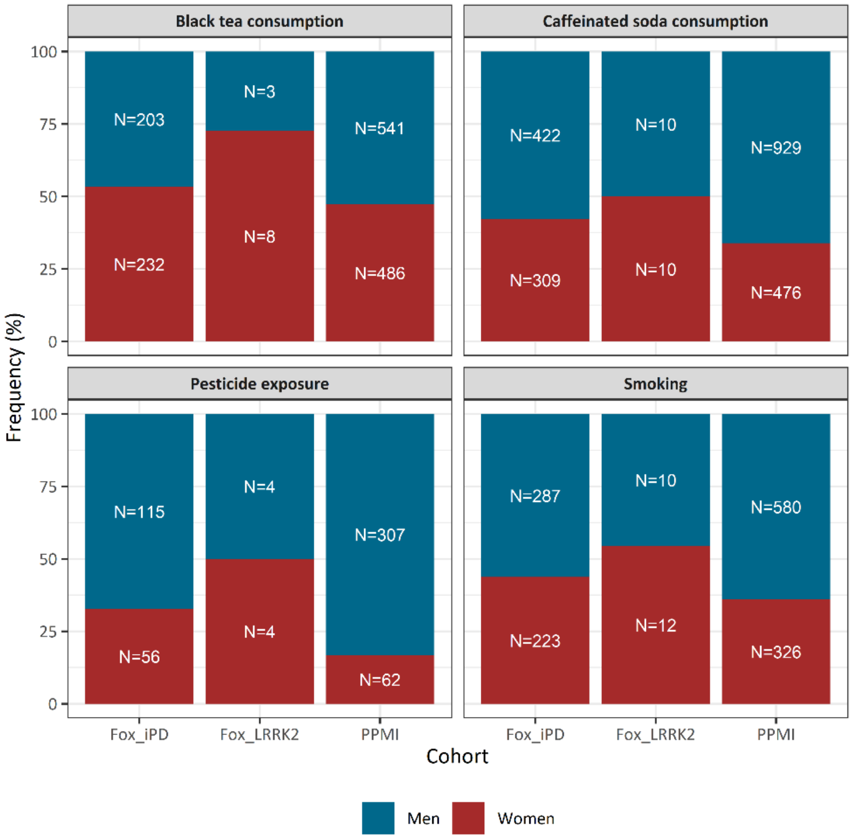

Supplementary Figure 1. Distribution of women and men across different environmental or lifestyle factors. The stacked bar plots display the increased fraction of men who were exposed to pesticides, smoked and consumed caffeinated soda and the more balanced fraction of men and women who drank black tea. The fraction of men (blue) and women (red) who consume black tea, caffeinated soda, smoked or were exposed to pesticides are shown. The distribution across the three analyzed cohorts is shown (i.e., PPMI-Online, Fox Insight (iPD) and Fox Insight (*LRRK2*-PD)). *N*=number of individuals.

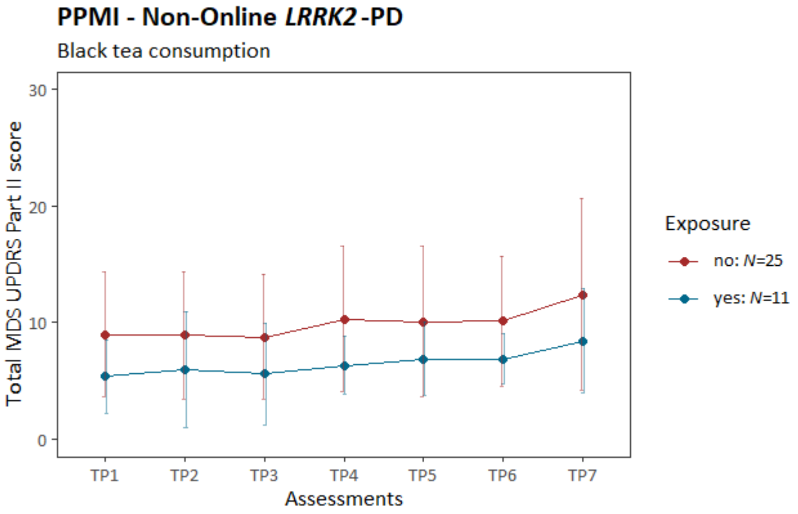

Supplementary Figure 2. Motor aspect severity over time stratified by black tea consumption.
The plot shows the progression of PD motor features along the longitudinal assessments. The mean cumulative MDS-UPDRS Part II score is indicated at each time period, and the error bars show the corresponding standard deviation. Patients with *LRRK2*-PD (PPMI participants that are not enrolled in the PPMI-Online study) are shown. The patients are stratified by black tea consumption. *LRRK2*-PD=Patients with PD that carry the LRRK2 p.Gly2019Ser variant, TP=Time period, *N*=number of individuals.

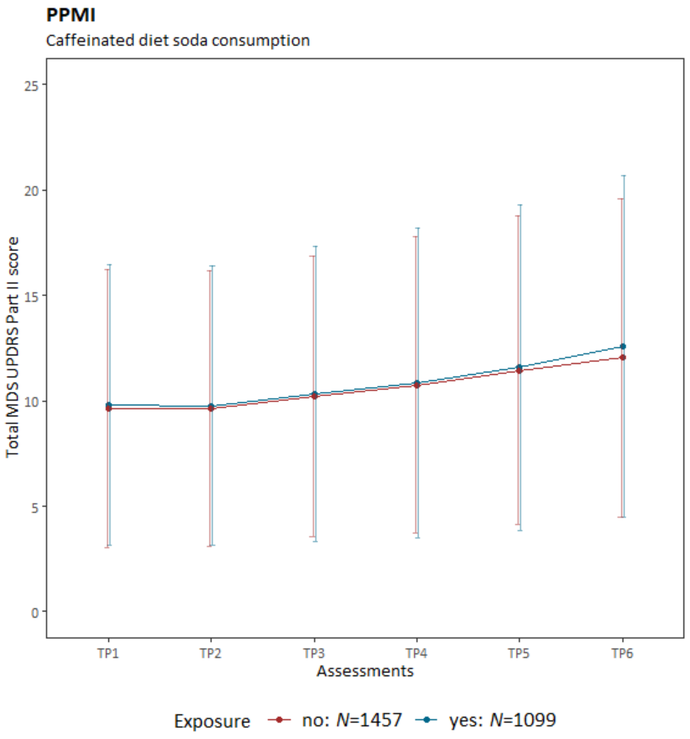

Supplementary Figure 3. Motor aspect severity over time stratified by caffeinated diet soda consumption. The plots show the progression of PD motor features along the longitudinal assessments. The mean cumulative MDS-UPDRS Part II score is indicated at each time period, and the error bars show the corresponding standard deviation. Patients with iPD (PPMI-Online) are shown. The patients are stratified by caffeinated diet soda consumption. TP=Time period. *N*=number of individuals.

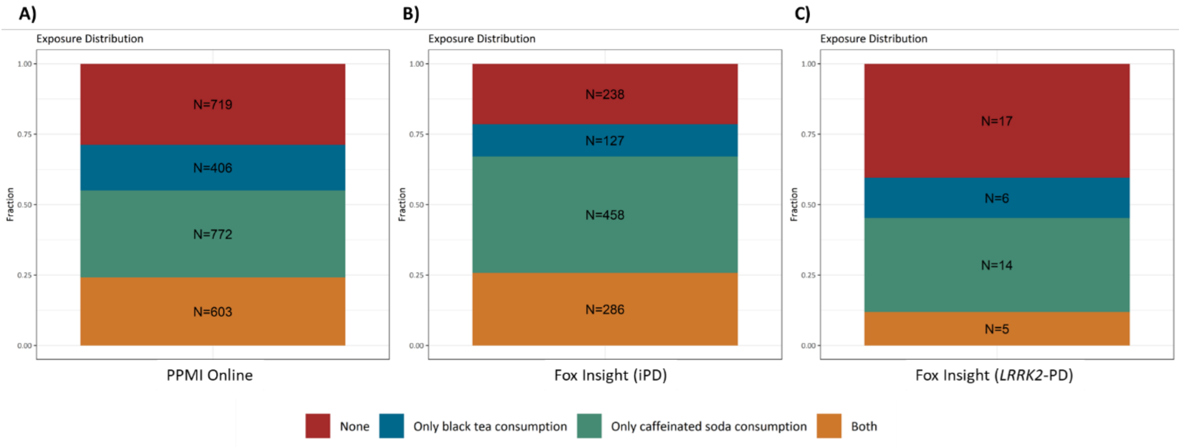

Supplementary Figure 4. Distribution of caffeinated beverages consumption. The stacked bar plots display the fraction of participants who consume black tea (blue), caffeinated soda (green), both beverages (orange) or none (red). There is no correlation between the two beverages, as a substantial fraction of participants consumed both beverages, only one or none. The distribution across the three analyzed cohorts is shown (i.e., PPMI-Online, Fox Insight (iPD) and Fox Insight (*LRRK2*-PD)). *N*=number of individuals.

A)

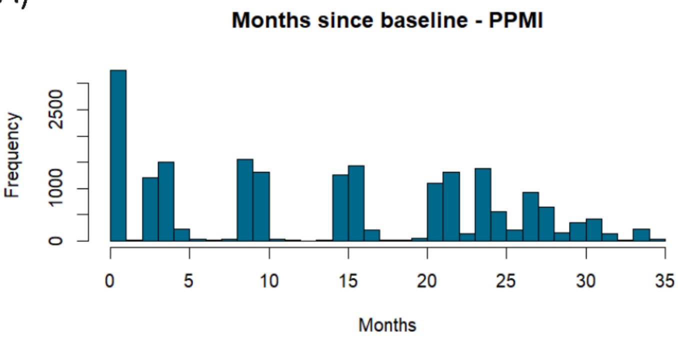

Time period	*N*
TP1 (baseline at 0 months)	2815
TP2 (0-6 months)	2673
TP3 (6-12 months)	2692
TP4 (12-18 months)	2670
TP5 (18-27 months)	2481
TP6 (27-35 months)	1160

B)

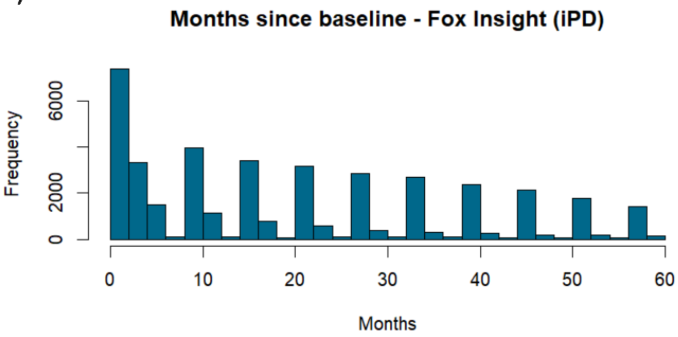

Time period	*N*
TP1 (baseline at 0 months)	2319
TP2 (0-10 months)	2103
TP3 (10-20 months)	2077
TP4 (20-30 months)	2121
TP5 (30-40 months)	1850
TP6 (40-50 months)	1267
TP7 (50-60 months)	1416

C)

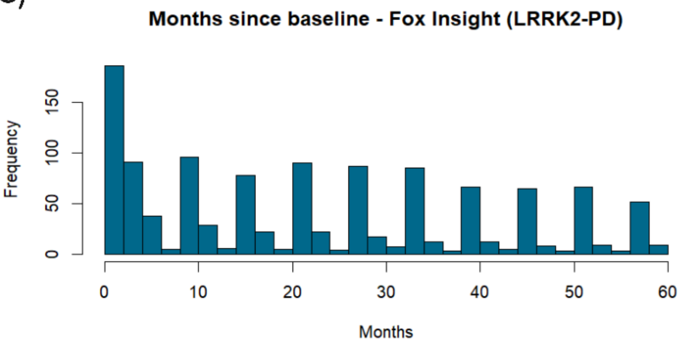

Time period-	*N*
TP1 (baseline at 0 months)	81
TP2 (0-10 months)	72
TP3 (10-20 months)	65
TP4 (20-30 months)	73
TP5 (30-40 months)	60
TP6 (40-50 months)	48
TP7 (50-60 months)	54

Supplementary Figure 5. Enrollment of Patients in the PPMI-Online and Fox Insight longitudinal cohort. The bar charts show the number of patients with PD that longitudinally responded to the motor aspects assessment in the months since baseline. The assessments were stratified into six time periods (PPMI-Online, **A**) or seven (Fox Insight iPD and *LRRK2*-PD, **B-C**). The number of patients per assessment is also presented in the corresponding tables. *N*=Number of individuals, iPD=idiopathic Parkinson's disease, *LRRK2*-PD= Patients with PD that carry the LRRK2 p.Gly2019Ser variant.

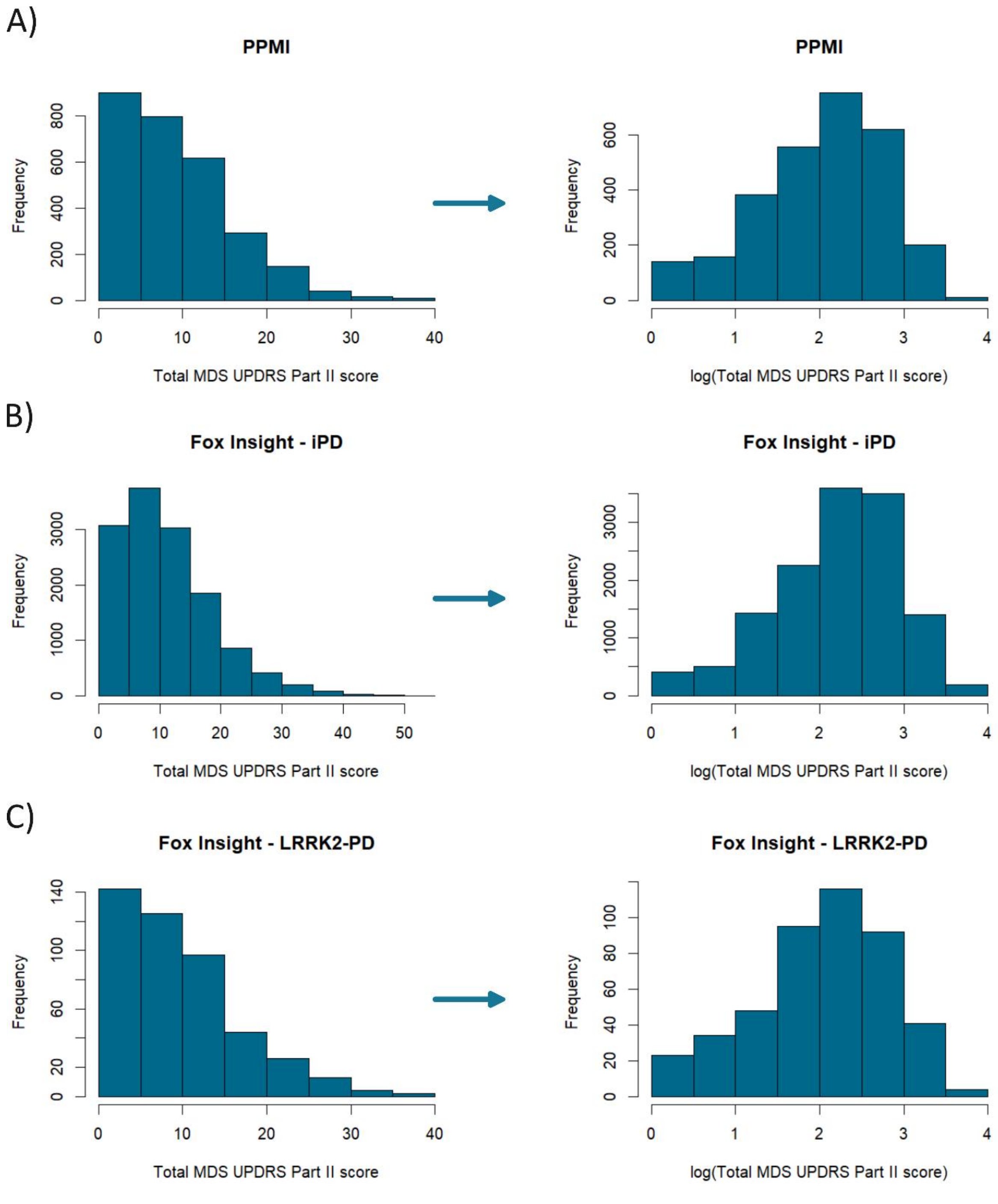

Supplementary Figure 6. Distribution of the motor aspect severity score.
The histograms show the frequency of the cumulative MDS-UPDRS Part II score. As there is no normal distribution the score has been logarithmized. The plots show the data of patients with iPD (PPMI-Online **A** and Fox Insight in **B**) or with *LRRK2*-PD (Fox Insight in **C**). iPD=idiopathic Parkinson's disease, *LRRK2*-PD=Patients with PD that carry the LRRK2 p.Gly2019Ser variant.

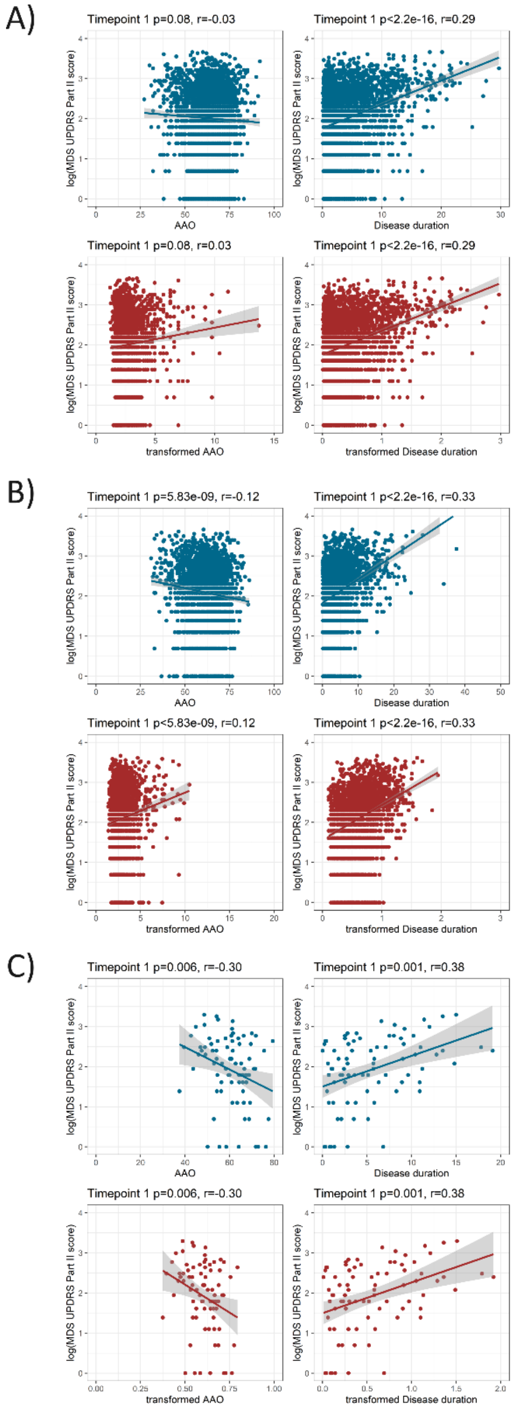

Supplementary Figure 7. Association between age at onset or disease duration and motor aspects score. The scatter plots show the relationship between the age of disease onset (AAO) and disease duration with the logarithmized MDS-UPDRS part II score. The untransformed data are shown in blue, and the transformed data in red (by using fractional polynomials). The plots show the data of patients with iPD (PPMI-Online **A** and Fox Insight in **B**) or with *LRRK2*-PD (Fox Insight in **C**). iPD=idiopathic Parkinson's disease, *LRRK2*-PD=Patients with PD that carry the LRRK2 p.Gly2019Ser variant, *r*=Spearman's rank correlation rho, *p*=Spearman's rank correlation *p*-value.

Supplementary Table 1. The association between coffee consumption and motor aspect severity over time. The motor aspect severity was evaluated longitudinally over time and assessed with a mixed linear model in the PPMI-Online and Fox Insight cohort.

	**Estimate**	***SE***	***p*-value**
**PPMI-Online (iPD: yes=2059, no=548)**			
**Time period 2**	-0.002	0.01	0.844
**Time period 3**	0.07	0.01	3.43×10^{-12}
**Time period 4**	0.12	0.01	$<2.00\times10^{-16}$
**Time period 5**	0.20	0.01	$<2.00\times10^{-16}$
**Time period 6**	0.26	0.01	$<2.00\times10^{-16}$
**Coffee**	0.05	0.03	0.157
**AAO**	-0.06	0.01	8.97×10^{-5}
**Disease duration**	0.63	0.04	$<2.00\times10^{-16}$
**Fox Insight (iPD: yes=1054, no=312)**			
**Time period 2**	0.02	0.01	0.218
**Time period 3**	0.08	0.01	1.740×10^{-8}
**Time period 4**	0.16	0.01	$<2.00\times10^{-16}$
**Time period 5**	0.23	0.02	$<2.00\times10^{-16}$
**Time period 6**	0.28	0.02	$<2.00\times10^{-16}$
**Time period 7**	0.32	0.02	$<2.00\times10^{-16}$
**Coffee**	0.01	0.04	0.782
**AAO**	-0.03	0.02	0.116
**Disease duration**	0.71	0.07	$<2.00\times10^{-16}$
**OFF-episodes**	0.24	0.04	9.030×10^{-11}
**Fox Insight (*LRRK2*-PD: yes=37, no=10)**			
**Time period 2**	0.01	0.08	0.935
**Time period 3**	0.06	0.08	0.404
**Time period 4**	0.20	0.08	0.010
**Time period 5**	0.18	0.08	0.020
**Time period 6**	0.34	0.08	4.51×10^{-5}
**Time period 7**	0.36	0.08	1.59×10^{-5}
**Coffee**	-0.04	0.23	0.851
**AAO**	-0.97	1.23	0.431
**Disease duration**	0.48	0.24	0.051
**OFF-episodes**	0.68	0.20	0.002

N=Number of individuals, iPD=idiopathic Parkinson's disease, *LRRK2*-PD= Patients with PD that carry the LRRK2 p.Gly2019Ser variant, Formula in R (package lme4): lmer(log(Cumulative MDS-UPDRS Part II Score) ~ Assessment time periods + mfp transformed AAO + mfp transformed disease duration + Environmental/Lifestyle factor (yes/no) + Experience of OFF episodes* + (1|Patient ID))
*If applicable
Baseline categories: Time period=Time period 1 (i.e., assessment at enrolment)

Supplementary Table 2. The association between green tea consumption and motor aspect severity over time. The motor aspect severity was evaluated longitudinally over time and assessed with a mixed linear model in the PPMI-Online and Fox Insight cohort.

	**Estimate**	***SE***	***p*-value**
**PPMI-Online (iPD: yes=385, no=2164)**			
**Time period 2**	-0.004	0.01	0.660
**Time period 3**	0.07	0.01	1.34×10^{-11}
**Time period 4**	0.12	0.01	$<2.00\times10^{-16}$
**Time period 5**	0.20	0.01	$<2.00\times10^{-16}$
**Time period 6**	0.26	0.01	$<2.00\times10^{-16}$
**Green tea**	0.04	0.04	0.360
**AAO**	-0.06	0.01	7.41×10^{-5}
**Disease duration**	0.62	0.04	$<2.00\times10^{-16}$
**Fox Insight (iPD: yes=189, no=943)**			
**Time period 2**	0.01	0.02	0.356
**Time period 3**	0.08	0.02	2.23×10^{-7}
**Time period 4**	0.16	0.02	$<2.00\times10^{-16}$
**Time period 5**	0.23	0.02	$<2.00\times10^{-16}$
**Time period 6**	0.28	0.02	$<2.00\times10^{-16}$
**Time period 7**	0.31	0.02	$<2.00\times10^{-16}$
**Green tea**	-0.03	0.05	0.557
**AAO**	-0.02	0.02	0.274
**Disease duration**	0.67	0.07	$<2.00\times10^{-16}$
**OFF-episodes**	0.25	0.04	3.18×10^{-10}
**Fox Insight (*LRRK2*-PD: yes=9, no=34)**			
**Time period 2**	-0.01	0.08	0.889
**Time period 3**	0.09	0.08	0.260
**Time period 4**	0.22	0.08	0.006
**Time period 5**	0.23	0.08	0.005
**Time period 6**	0.39	0.09	1.19×10^{-5}
**Time period 7**	0.40	0.08	4.65×10^{-6}
**Green tea**	-0.23	0.25	0.358
**AAO**	-0.65	1.32	0.625
**Disease duration**	0.45	0.25	0.080
**OFF-episodes**	0.54	0.22	0.018

N=Number of individuals, iPD=idiopathic Parkinson's disease, *LRRK2*-PD= Patients with PD that carry the LRRK2 p.Gly2019Ser variant, Formula in R (package lme4): lmer(log(Cumulative MDS-UPDRS Part II Score) ~ Assessment time periods + mfp transformed AAO + mfp transformed disease duration + Environmental/Lifestyle factor (yes/no) + Experience of OFF episodes* + (1|Patient ID))

*If applicable

Baseline categories: Time period=Time period 1 (i.e., assessment at enrolment)

Supplementary Table 3A. The association between pesticide exposure and motor aspect severity over time. The motor aspect severity was evaluated longitudinally over time and assessed with a linear mixed model in the PPMI-Online and Fox Insight cohort.

	**Estimate**	***SE***	***p*-value**
**PPMI-Online (iPD: yes=369, no=2098)**			
**Time period 2**	0.001	0.01	0.891
**Time period 3**	0.07	0.01	4.18×10^{-13}
**Time period 4**	0.12	0.01	$<2.00\times10^{-16}$
**Time period 5**	0.20	0.01	$<2.00\times10^{-16}$
**Time period 6**	0.27	0.01	$<2.00\times10^{-16}$
**Pesticide exposure**	0.19	0.04	1.24×10^{-6}
**AAO**	-0.06	0.01	1.48×10^{-4}
**Disease duration**	0.64	0.04	$<2.00\times10^{-16}$
**Sex: Men**	0.14	0.03	9.94×10^{-7}
**Fox Insight (iPD: yes=171, no=630)**			
**Time period 2**	0.01	0.02	0.610
**Time period 3**	0.09	0.02	1.350×10^{-05}
**Time period 4**	0.15	0.02	$5.510E^{-14}$
**Time period 5**	0.22	0.02	$<2.00\times10^{-16}$
**Time period 6**	0.27	0.02	$<2.00\times10^{-16}$
**Time period 7**	0.30	0.02	$<2.00\times10^{-16}$
**Pesticide exposure**	0.08	0.06	0.146
**AAO**	-0.02	0.02	0.484
**Disease duration**	0.76	0.09	$<2.00\times10^{-16}$
**OFF-episodes**	0.25	0.05	2.150×10^{-7}
**Sex: Men**	0.18	0.05	1.590×10^{-4}

N=Number of individuals, iPD=idiopathic Parkinson's disease, Formula in R (package lme4): lmer(log(Cumulative MDS-UPDRS Part II Score) ~ Assessment time periods + mfp transformed AAO + mfp transformed disease duration + Environmental/Lifestyle factor (yes/no) + Experience of OFF episodes* + Sex + (1|Patient ID))
*If applicable, Tested for significance at α = 0.017
Baseline categories: Time period=Time period 1 (i.e., assessment at enrolment), Sex=Women

Supplementary Table 3B. The association between smoking and motor aspect severity over time. The severity of motor aspect was evaluated longitudinally over time and assessed using a linear mixed model in the PPMI-Online.

	**Estimate**	***SE***	***p*-value**
**PPMI-Online (iPD: yes=906, no=1670)**			
**Time period 2**	0.001	0.01	0.911
**Time period 3**	0.07	0.01	1.970×10^{-11}
**Time period 4**	0.12	0.01	$<2.00\times10^{-16}$
**Time period 5**	0.20	0.01	$<2.00\times10^{-16}$
**Time period 6**	0.26	0.01	$<2.00\times10^{-16}$
**Smoking**	0.12	0.03	3.170×10^{-5}
**AAO**	-0.05	0.01	0.001
**Disease duration**	0.64	0.04	$<2.00\times10^{-16}$
**Sex: Men**	0.17	0.03	8.620×10^{-10}

N=Number of individuals, iPD=idiopathic Parkinson's disease, Formula in R (package lme4): lmer(log(Cumulative MDS-UPDRS Part II Score) ~ Assessment time periods + mfp transformed AAO + mfp transformed disease duration + Environmental/Lifestyle factor (yes/no) + Experience of OFF episodes* + Sex + (1|Patient ID))
*If applicable
Baseline categories: Time period=Time period 1 (i.e., assessment at enrolment), Sex=Women

Supplementary Table 3C. The association between black tea consumption and motor aspect severity over time. The motor aspect severity was evaluated longitudinally over time and assessed using a linear mixed model in the Fox Insight cohort (*LRRK2*-PD)

	**Estimate**	***SE***	***p*-value**
**Fox Insight (*LRRK2*-PD: yes=11, no=31)**			
**Time period 2**	-0.01	0.08	0.855
**Time period 3**	0.09	0.08	0.296
**Time period 4**	0.22	0.08	0.006
**Time period 5**	0.23	0.08	0.006
**Time period 6**	0.39	0.09	1.420×10^{-5}
**Time period 7**	0.40	0.09	5.830×10^{-6}
**Black tea**	-0.53	0.23	0.028
**AAO**	-0.90	1.31	0.495
**Disease duration**	0.37	0.24	0.138
**OFF-episodes**	0.58	0.21	0.009
**Sex: Men**	-0.08	0.21	0.718

N=Number of individuals, *LRRK2*-PD= Patients with PD that carry the LRRK2 p.Gly2019Ser variant, Formula in R (package lme4): lmer(log(Cumulative MDS-UPDRS Part II Score) ~ Assessment time periods + mfp transformed AAO + mfp transformed disease duration + Environmental/Lifestyle factor (yes/no) + Experience of OFF episodes* + Sex + (1|Patient ID))

*If applicable

Baseline categories: Time period=Time period 1 (i.e., assessment at enrolment), Sex=Women

Supplementary Table 3D. The association between caffeinated soda consumption and motor aspect severity over time. The motor aspect severity was evaluated longitudinally over time and assessed with a linear mixed model in the PPMI-Online and Fox Insight cohort.

	**Estimate**	***SE***	***p*-value**
**PPMI-Online (iPD: yes=1405, no=1154)**			
**Time period 2**	0.0004	0.01	0.966
**Time period 3**	0.07	0.01	1.180×10^{-12}
**Time period 4**	0.12	0.01	$<2.00\times10^{-16}$
**Time period 5**	0.20	0.01	$<2.00\times10^{-16}$
**Time period 6**	0.26	0.01	$<2.00\times10^{-16}$
**Caffeinated soda**	0.13	0.03	2.080×10^{-6}
**AAO**	-0.06	0.01	2.600×10^{-5}
**Disease duration**	0.64	0.04	$<2.00\times10^{-16}$
**Sex: Men**	0.17	0.03	1.520×10^{-9}
**Fox Insight (iPD: yes=731, no=357)**			
**Time period 2**	0.01	0.02	0.751
**Time period 3**	0.08	0.02	4.490×10^{-6}
**Time period 4**	0.15	0.02	$<2.00\times10^{-16}$
**Time period 5**	0.23	0.02	$<2.00\times10^{-16}$
**Time period 6**	0.28	0.02	$<2.00\times10^{-16}$
**Time period 7**	0.31	0.02	$<2.00\times10^{-16}$
**Caffeinated soda**	0.07	0.04	0.119
**AAO**	-0.02	0.02	0.220
**Disease duration**	0.72	0.07	$<2.00\times10^{-16}$
**OFF-episodes**	0.25	0.04	4.920×10^{-10}
**Sex: Men**	0.20	0.04	2.040×10^{-7}
**Fox Insight (*LRRK2*-PD: yes=20, no=23)**			
**Time period 2**	-0.01	0.08	0.881
**Time period 3**	0.09	0.08	0.259
**Time period 4**	0.22	0.08	0.006
**Time period 5**	0.23	0.08	0.005
**Time period 6**	0.39	0.09	1.160×10^{-5}
**Time period 7**	0.40	0.08	4.890×10^{-6}
**Caffeinated soda**	0.35	0.22	0.111
**AAO**	-1.18	1.37	0.394
**Disease duration**	0.46	0.25	0.069
**OFF-episodes**	0.42	0.23	0.080
**Sex: Men**	0.07	0.21	0.749

N=Number of individuals, iPD=idiopathic Parkinson's disease, *LRRK2*-PD= Patients with PD that carry the LRRK2 p.Gly2019Ser variant, Formula in R (package lme4): lmer(log(Cumulative MDS-UPDRS Part II Score) ~ Assessment time periods + mfp transformed AAO + mfp transformed disease duration + Environmental/Lifestyle factor (yes/no) + Experience of OFF episodes* + Sex +(1|Patient ID))

*If applicable

Baseline categories: Time period=Time period 1 (i.e., assessment at enrolment), Sex=Women

Supplementary Table 4. The association between black tea consumption and motor aspect severity over time. The motor aspect severity was evaluated longitudinally over time and assessed with a mixed linear model in patients with *LRRK2*-PD PPMI participants who are not enrolled in the PPMI-Online study.

	**Estimate**	***SE***	***p*-value**
**PPMI – Non-Online (iPD: yes=11, no=25)**			
**Time period 2**	-0.04	0.11	0.735
**Time period 3**	-0.01	0.11	0.948
**Time period 4**	0.27	0.12	0.019
**Time period 5**	0.28	0.12	0.018
**Time period 6**	0.35	0.12	0.004
**Time period 7**	0.40	0.12	0.001
**Black tea**	-0.41	0.18	0.026
**AAO**	-0.51	1.16	0.665
**Disease duration**	0.14	0.04	0.001

N=Number of individuals, iPD=idiopathic Parkinson's disease, *LRRK2*-PD= Patients with PD that carry the LRRK2 p.Gly2019Ser variant, Formula in R (package lme4): lmer(log(Cumulative MDS-UPDRS Part II Score) ~ Assessment time periods + mfp transformed AAO + mfp transformed disease duration + Environmental/Lifestyle factor (yes/no) + Experience of OFF episodes* + (1|Patient ID))
*If applicable
Baseline categories: Time period=Time period 1 (i.e., assessment at enrolment)

Supplementary Table 5. Overview of patients with iPD and *LRRK2*-PD that are also affected by type 2 diabetes (T2DM), stratified by caffeinated soda consumption within Fox Insight.

**PD subtype**	**Caffeinated soda**	**T2DM (N)**	**No T2DM (N)**	**NA (N)**
**iPD**	**yes**	16	217	609
	**no**	8	11	283
***LRRK2*-PD**	**yes**	1	4	8
	**no**	0	8	12

N=Number of individuals, iPD=idiopathic Parkinson's disease, *LRRK2*-PD= Patients with PD that carry the LRRK2 p.Gly2019Ser variant, NA=Information not available
